# Chromatin assembly factor 1 subunit A promotes TLS pathway by recruiting E3 ubiquitin ligase RAD18 in cancer cells

**DOI:** 10.1038/s41419-025-07468-5

**Published:** 2025-03-01

**Authors:** Bing Wen, Hai-Xiang Zheng, Jing-Hua Heng, Qian Tang, Dan-Xia Deng, Zhi-Da Zhang, Lian-Di Liao, Li-Yan Xu, En-Min Li

**Affiliations:** 1 The Key Laboratory of Molecular Biology for the High Cancer Incidence Coastal Chaoshan Area, Department of Biochemistry and Molecular Biology, Shantou University Medical College, Shantou, 515041 Guangdong P.R. China; 2 Chaoshan Branch of State Key Laboratory for Esophageal Cancer Prevention and Treatment, Institute of Oncologic Pathology, Shantou University Medical College, Shantou, 515041 Guangdong P.R. China; 3 The Laboratory for Cancer Molecular Biology, Shantou Academy of Medical Sciences, Shantou, 515041 Guangdong P.R. China

**Keywords:** Ubiquitylation, Translesion synthesis

## Abstract

The translesion DNA synthesis (TLS) pathway mediated by proliferating cell nuclear antigen (PCNA) monoubiquitination is an essential mechanism by which cancer cells bypass DNA damage caused by DNA damage to maintain genomic stability and cell survival. Chromatin assembly factor 1 subunit A (CHAF1A) traditionally promotes histone assembly during DNA replication. Here, we revealed that CHAF1A is a novel regulator of the TLS pathway in cancer cells. CHAF1A promotes restart and elongation of the replication fork under DNA replication stress. Mechanistically, the C-terminal domain of CHAF1A directly interacts with E3 ubiquitin ligase RAD18, enhancing RAD18 binding on the stalled replication fork. CHAF1A facilitates PCNA K164 monoubiquitination mediated by RAD18, thereby promoting the recruitment of Y-family DNA polymerases and enhancing cancer cell resistance to DNA damage. In addition, CHAF1A-mediated RAD18 recruitment and PCNA monoubiquitination are independent of the CHAF1A-PCNA interaction and its histone assembly function. Taken together, these findings improve our understanding of the mechanisms that regulate the TLS pathway and provide insights into the relationship between CHAF1A and DNA replication stress in cancer cells.

## Highlights

- CHAF1A promotes UV-mediated PCNA K164 monoubiquitination in cancer cells.
- CHAF1A is a new regulator of translesion DNA synthesis pathway in cancer cells.
- CHAF1A accelerates RAD18 recruitment in the stalled replication forks by directly interacting with RAD18.

## Introduction

The faithful transmission of genetic information to daughter cells is essential for maintaining the genomic integrity in normal cells and depends on complete and accurate DNA replication during the S-phase of cell division [1]. Due to the accumulation of DNA damage, genomic instability is a major hallmark of various cancers. Based on this characteristic of cancer, radiotherapy and chemotherapy have become the main methods of cancer treatment. However, DNA damage tolerance (DDT) pathway of cancer cells leads to the resistance of cancer cells to radiotherapy and chemotherapy [2]. However, endogenous and exogenous stimuli can cause DNA lesions that prevent accurate DNA replication, causing DNA replication stress [3, 4]. Since the correct processing of stalled or damaged replication forks is critical for cell survival [5], cancer cells have developed mechanisms to resist DNA replication stress and promote DNA repair to combat DNA damage [6].

In mammalian cells, the predominant mechanism used to bypass DNA replication stress is the translesion synthesis (TLS) pathway, which is mediated by monoubiquitinated PCNA [7]. Shortening of the nucleotide pool (HU), DNA adducts (UV, MMS), etc., uncouple the replicative polymerase to the MCM helicase complex, causing DNA replication stress, which then activates PCNA monoubiquitination [8]. PCNA monoubiquitination at Lys164 (K164) is mediated by the RAD6/RAD18 complex [9–15]. Exposure to UV generates two types of pyrimidine dimers: cyclobutane pyrimidine dimer (CPD) and pyrimidine (6-4) pyrimidone (6-4PP). Because the damage type is clear, UV is often used as an inducer in the study of TLS pathway. In addition, PCNA K164 monoubiquitination promotes the conversion of replicative DNA polymerase Pol δ/ε into damage-tolerant translesion DNA polymerases, such as Pol η, Pol ι, Pol κ [16], or REV1 [17]. TLS pathway activation is therefore beneficial for cancer cell survival under DNA replication stress and is closely related to the malignant progression of cancers and resistance to chemotherapy [18, 19].

Chromatin assembly factor 1 subunit A (CHAF1A, also known as p150 or CAF1A) is the largest subunit of the CAF-1 complex. CAF-1 is a heterotrimeric histone chaperone complex that consists of three subunits: CHAF1A, chromatin assembly factor 1 subunit B (CHAF1B, also known as p60 or CAF1B), and histone-binding protein RBBP4 (also known as p48) [20]. CAF-1 mainly binds to newly synthesized histones (H3 especially H3.1/H3.2, and H4) by interacting with PCNA and promotes their deposition at the replication fork during S-phase. Previously, we found that co-high expression of CHAF1A and PCNA is a poor prognostic factor for patients with esophageal cancer [21]. Although the synergistic role of CHAF1A and PCNA in normal DNA replication has been widely reported, their role under TLS pathway has been unclear.

Here, we demonstrate a novel function of CHAF1A in regulating PCNA K164 monoubiquitination mediated-TLS pathway in cancer cells. We found that CHAF1A directly interacts with RAD18 to mediate the loading of RAD18 onto the stalled replication fork and enhance the monoubiquitination of PCNA at K164 site.

## Materials and methods

### Cell culture

HEK293T cell line and lung cancer cell line A549 were purchased from American Type Culture Collection (ATCC, Manassas, VA, USA). We obtained the human esophageal cancer cell lines KYSE150 and KYSE510 that were established by Dr. Shimada Yutaka (Faculty of Medicine, Kyoto University, Kyoto, Japan) [22]. Lung normal cell line MRC-5 was procured from the Cell Bank of Chinese Academy of Sciences (Shanghai, China). The immortalized human esophageal epithelial cell line NE3 was donated by Professor Sai-Wah Tsao (Department of Anatomy, University of Hong Kong, Hong Kong, China). NE3 cells were cultured in a 1:1 mixture of defined keratinocyte serum-free medium (dKSFM; Gibco; Thermo Fisher Scientific, Inc., Waltham, MA, USA) and EpiLife medium (Cascade Biologics, Inc., Portland, OR, USA) in 5% CO_2_ at 37 °C. MRC-5 cells and HEK293T cells were cultured in DMEM supplemented with 10% fetal bovine serum (FBS), penicillin (100 mg/mL), and streptomycin (100 mg/mL). KYSE150, A549 and KYSE510 cells were cultured in RPMI-1640 medium supplemented with 10% FBS, penicillin (100 mg/mL), and streptomycin (100 mg/mL). All cell lines were cultured in a 5% CO_2_ atmosphere at 37 °C. All cells were tested for mycoplasma contamination using short tandem repeat (STR) analysis (IGE bio, Guangzhou, China).

### Generation of cell lines with inducible CHAF1A knockdown

Cells with inducible CHAF1A knockdown were generated using a lentiviral system [23]. First, we purchased shCHAF1A oligos (shCHAF1A#1 oligo1: 5ʹ-CCG GCC GAC TCA ATT CCT GTG TAA ACT CGA GTT TAC ACA GGA ATT GAG TCG GTT TTT G-3ʹ, shCHAF1A#1 oligo2: 5ʹ- AAT TCA AAA ACC GAC TCA ATT CCT GTG TAA ACT CGA GTT TAC ACA GGA ATT GAG TCG G-3ʹ; shCHAF1A#2 oligo1: 5ʹ-CCG GGA TAC TTG AAC CGA CTC AAT TCT CGA GAA TTG AGT CGG TTC AAG TAT CTT TTT G -3ʹ, shCHAF1A#2 oligo2: 5ʹ- AAT TCA AAA AGA TAC TTG AAC CGA CTC AAT TCT CGA GAA TTG AGT CGG TTC AAG TAT C-3ʹ) from Gene Pharma (Shanghai, China), then transferred the oligos into a single inducible lentiviral vector (pLKO-TET-Puro, cleaved by A*ge* I) to create pLKO-TET-Puro-shCHAF1A#1 and pLKO-TET-Puro-shCHAF1A#2 expression vector. The targeting sequences of shCHAF1A#1 and shCHAF1A#2 were both in the 3’ UTR of CHAF1A mRNA, which was conducive to the recovery of exogenous CHAF1A. We used the pLKO-TET-Puro-scramble vector containing scramble shRNA as a negative control vector. Next, HEK293T cells were co-transfected with the expression vector (pLKO-TET-Puro-scramble, pLKO-TET-Puro-shCHAF1A#1, and pLKO-TET-Puro-shCHAF1A#2) and the packaging vector (psPAX2 and pMD2.G), and the lentiviral supernatant was collected and infected into cancer cells 48 h later. Finally, we successfully generated doxycycline (DOX) -inducible CHAF1A knockdown in the human cancer cell lines A549 and KYSE510. Cells were treated with 1 μg/μl DOX for 48 h to knock down endogenous CHAF1A. In some experiments, two pooled shRNAs were used.

### Plasmids, siRNAs, and chemicals

Cells were transfected with plasmids and siRNAs using Lipofectamine 3000 (L3000015, Thermo Fisher Scientific, Waltham, MA, USA) and Lipofectamine RNAiMAX (13778150, Thermo Fisher Scientific), respectively, using standard protocols. Full-length cDNA clones encoding human PCNA were extracted from HEK293T cells, amplified using PCR, and subcloned into pCMV-Flag-N vector and pCMV-HA-N vector. Full-length cDNA clones encoding human RAD6A were extracted from HEK293T cells, amplified using PCR, and subcloned into pcDNA3.1-HA vector. pET28a-RPA1 vector (P65534) is purchased from MIAOLING BIO (Wuhan, China). Full-length cDNA clones encoding human RPA2 and RPA3 were extracted from HEK293T cells, amplified using PCR, and subcloned into pET32a vector. Full-length cDNA clone encoding human RAD18 was extracted from HEK293T cells, amplified using PCR, and subcloned into pGEX-6P-1 vector. The cDNA clones of human CHAF1A wild-type (WT), N-terminal (N), middle domain (M), and C-terminal (C) and PIP domain-truncated mutations were extracted from HEK293T cells, amplified using PCR, and subcloned into pET32a or pEGFP-C1 vectors. siRNAs targeting CHAF1A (siCHAF1A#1: 5ʹ-CAG CCA UGG AUU GCA AAG A-3ʹ, siCHAF1A#2: 5ʹ-CAG AAC GAC AAG UUG GCA U-3ʹ, and siCHAF1A#3: 5ʹ-CUC CGC AGA AUA ACU AAG A-3ʹ) and siRNAs targeting RAD18 (siRAD18#1: 5ʹ- GGU AGA CUC UUU GGC ACU U-3ʹ, siRAD18#2: 5ʹ- GCC CGA GGU UAA UGU AGU U-3ʹ, and siRAD18#3: 5ʹ- GCA GUG AUG CUU AUG GUU U-3ʹ) were purchased from Gene Pharma (Shanghai, China). Three pooled siRNAs were used in all experiments. Doxycycline (T1687) was purchased from TOPSCIENCE (Shanghai, China). Hydroxyurea (H8627) was purchased from Sigma-Aldrich (St. Louis, MO, USA). Cisplatin (S1166) was purchased from Selleck (USA).

### Western blotting

Western blotting analysis was performed using standard protocols, as described previously [24]. Briefly, samples were heated at 95 °C for 5–15 min in 1 × sodium dodecyl sulfate (SDS) loading buffer, subjected to electrophoresis in 10% SDS-polyacrylamide gels, and transferred to polyvinylidene difluoride (PVDF) membranes. Blocking and antibody dilution were performed using 5% nonfat milk (or bovine serum albumin, BSA) in phosphate-buffered saline (PBS). After incubation with primary antibodies at 4 °C overnight and secondary antibodies at 25 °C for 1.5 h, the membranes were washed three times in 1× PBST (1× PBS with 0.1% Tween-20). Images were acquired using a Chemodoc MP system (Bio-Rad, Hercules, CA, USA) with ImageLab software. The following antibodies were used: rabbit polyclonal anti-PCNA (10205-2-AP, Proteintech, Rosemont, USA; 1:5000), mouse monoclonal anti-PCNA (sc-56, Santa Cruz Biotechnology, Dallas, TX, USA; 1:1000), rabbit monoclonal anti-Ub-PCNA (K164) (13439S, Cell Signaling Technology, Danvers, MA, USA; 1:1000), mouse monoclonal anti-GFP-tag (sc-9996,, Santa Cruz Biotechnology; 1:1000), rabbit polyclonal anti-HA-tag (51064-2-AP, Proteintech; 1:8000), mouse monoclonal anti-Lamin B1 (66095-1-Ig, Proteintech; 1:10000), mouse monoclonal anti-CHAF1A (sc-133105, Santa Cruz Biotechnology; 1:1000), rabbit polyclonal anti-CHAF1A (17037-1-AP, Proteintech; 1:1000), rabbit polyclonal anti-RAD18 (18333-1-AP, Proteintech; 1:2000), mouse monoclonal anti-Pol ι (sc-101026, Santa Cruz Biotechnology; 1:1000), mouse monoclonal anti-Pol κ (sc-166667, Santa Cruz Biotechnology; 1:1000), mouse monoclonal anti-REV1 (sc-393022, Santa Cruz Biotechnology; 1:1000), mouse monoclonal anti-Pol η (sc-17770, Santa Cruz Biotechnology; 1:1000), mouse monoclonal anti-His Antibody (HT501, TransGen Biotech; 1:5000), mouse monoclonal anti-CHK1 (60277, Proteintech; 1:2000), rabbit monoclonal anti-phospho-CHK1 (Ser345) (2348S, Cell Signaling Technology; 1:1000), mouse monoclonal anti-RPA2 (ab2175, Abcam; 1:1000), rabbit monoclonal anti-phospho RPA2 (Ser8) (54762S, Cell Signaling Technology; 1:1000), horseradish peroxidase (HRP)-conjugated beta actin monoclonal antibody (HRP-60008, Proteintech; 1:10000), and HRP-conjugated monoclonal alpha tubulin (HRP-66031, Proteintech; 1:10000). Secondary antibodies included goat anti-mouse IgG-HRP (sc-2005, Santa Cruz; 1:5000) and goat anti-rabbit IgG-HRP antibodies (sc-2030, Santa Cruz Biotechnology; 1:5000).

### CoIP assays

Co immunoprecipitation (CoIP) was performed as described previously [25]. To detect interactions between endogenous proteins, Cells were lysed with IP buffer (50 mM Tris-HCl pH 7.5, 150 mM NaCl, and 0.5% NP-40) supplemented with a protease inhibitor cocktail (HY-K0010, MCE, Shanghai, China). After sonication and centrifugation at 12 000 rpm at 4 °C for 10 min, the protein concentration of the cell lysates was measured using a Pierce BCA Protein Assay Kit (23225, Thermo Fisher Scientific). Equal amounts of total protein (1 mg) were immunoprecipitated by incubation with protein A/G magnetic beads (HY-K0202, MCE) and indicated antibodies at 4 °C overnight with gentle rocking. To detect interactions between PCNA and exogenous GFP-CHAF1A (WT, ΔPIP1, and ΔPIP2), HEK293T cells were transfected with pEGFP-CHAF1A-WT, pEGFP-CHAF1A-ΔPIP1, pEGFP-CHAF1A-ΔPIP2, or pEGFP-vector plasmids, and total protein was collected after 48 h. Equal amounts of total protein (1 mg) were immunoprecipitated by incubation with protein A/G magnetic beads (HY-K0202, MCE) and anti-GFP antibody (sc-9996, Santa Cruz Biotechnology; 1:50) at 4 °C overnight with gentle rocking. The immunoprecipitated proteins were washed three times with cold IP buffer and denatured with 1× SDS sample buffer.

### Chromatin fractionation

The chromatin fractionation experiments were performed as described previously [26], with slight modifications. Cells in 10 cm dishes were digested using trypsin, collected by centrifugation at 500 × g for 5 min, washed with PBS, and lysed on ice in 600 μL of hypotonic buffer (10 mM Tris-HCl, pH 7.5, 2 mM MgCl_2_, 3 mM CaCl_2_, 320 mM sucrose, 1 mM dithiothreitol (DTT), and 0.3% NP40) supplemented with protease inhibitor cocktail (HY-K0010, MCE). After 10 min, the cells were centrifuged for 5 min at 2800 × *g*. The pellet was washed three times in hypotonic buffer, incubated with 100 μL nuclear extraction buffer (20 mM 2-[4-(2-hydroxyethyl) piperazin-1-yl] ethanesulfonic acid (HEPES), pH 7.7, 1.5 mM MgCl_2_, 420 mM NaCl, 200 mM ethylene diamine tetraacetic acid (EDTA), 25% glycerol, and 1 mM DTT) supplemented with a protease inhibitor cocktail (HY-K0010, MCE) for 30 min at 4 °C, and centrifuged at 8 000 × *g* for 15 min. The chromatin pellet was resuspended in 1× SDS loading buffer, sonicated, and subjected to western blot analysis.

### DNA fiber assays

The DNA fiber assay was performed as described previously [27]. For monitoring replication fork progression under HU treatment, cells were first incubated with 25 μΜ CldU (C6891, Sigma-Aldrich) for 30 min and then with 250 μM IdU (I7125, Sigma-Aldrich) in the presence of 50 μΜ HU for 30 min. Alternatively, the cells were labeled with CldU for 30 min, exposed to 2 mM HU for 2 h, and then incubated with IdU for 30 min before being harvested in PBS. For monitoring replication fork progression under UV radiation, cells were pulse-labeled with 25 μM IdU for 20 min. Cells were then washed with PBS buffer twice and radiated with UV (100 J/m^2^). After UV radiation, cells were labeled with 250 μM CldU for 40 min. Labeled cells were quickly trypsinized, resuspended in ice-cold PBS at a density of approximately 1 × 10^6^ cells/mL, and spotted onto a pre-cleaned glass slide (2.5 μL) for lysis in 7.5 μL of lysis buffer (0.5% SDS in 200 mM Tris-HCl (pH 7.4) with 50 mM EDTA). After 5 min, the slides were tilted at 25 °C relative to the horizontal and the resulting DNA spreads were air-dried and fixed in 3:1 methanol/acetic acid for 20 min. After rehydration in PBS, the samples were denatured in 2.5 M HCl for 1 h, washed with PBS, and blocked with 2% BSA in PBS (w/v) containing 0.1% Tween-20 for 40 min. Immunodetection was performed using the following primary antibodies: anti-CldU (rat monoclonal anti-5-bromo-2-deoxyuridine (BrdU)/CldU; BU1/75 ICR1, Abcam, Cambridge, UK; 1:200) and anti-IdU (mouse monoclonal anti-BrdU/IdU; clone B44, BD Biosciences, NJ, USA; 1:25). The samples were then incubated with the following secondary antibodies in a humidified chamber for 1 h at 25 °C: Alexa Fluor® 647 AffiniPure donkey anti-mouse (715-605-150, Jackson ImmunoResearch, West Grove, PA, USA; 1:200) or goat anti-rat Alexa Fluor 488 (A-11006, Invitrogen, San Francisco, CA, USA; 1:200). Images were acquired using a Zeiss (Jena, Germany) LSM 800 fluorescence microscope at 40× magnification and analyzed using ImageJ software (version 1.52). A minimum of 100 individual fibers were analyzed per experiment. Statistical analyses were performed using Prism (version 8.0.2, GraphPad).

### PLA assays

PLA assays was performed using Duolink PLA technology (Sigma-Aldrich) according to the manufacturer’s instructions. Cells were washed once with 1× PBS and treated with cytoskeletal (CSK) extraction buffer [0.2% Triton X-100, 20 mM HEPES-KOH (pH 7.9), 100 mM NaCl, 3 mM MgCl_2_, 300 mM sucrose, 1 mM ethylenebis (oxyethylenenitrilo) tetraacetic acid (EGTA)] containing 4% formaldehyde at 25 °C for 10 min. The cells were then washed three times with 1× PBS, permeabilized with 0.5% NP-40 in 1× PBS for 5 min, and blocked with 5% BSA in PBS at 25 °C for 1 h. Alternatively, to detect the interaction between RAD18 and EdU, cells were first labeled with 10 μM EdU for 15 min at 37 °C, and then subjected to a 100 J/m^2^ UV treatment for 1 h prior to two washes with PBS. Cells treated with CSK extraction buffer containing 4% formaldehyde at 25 °C for 10 min. The cells were then washed three times with 1× PBS, permeabilized with 0.5% NP-40 in 1× PBS for 5 min, and blocked with 5% BSA in PBS at 25 °C for 1 h. Then, cells were subjected to Click-iT reaction to attach biotin to EdU and incubated with the two relevant primary antibodies at 4 °C overnight. After incubation with the indicated primary antibodies at 4 °C overnight, the cells were washed three times with 1× PBS and incubated with anti-mouse and anti-rabbit plus PLA probes (PLA kit, Sigma-Aldrich) at 37 °C for 1 h. The PLA reaction was performed using Duolink in Situ Detection Reagents (PLA kit) according to the manufacturer’s instructions. Finally, the cells were washed three times with buffer B, stained with 4’,6-diamidino-2-phenylindole (DAPI) during the second wash, and mounted with antifade mounting medium (Beyotime) on slides that were sealed with nail polish. Images were captured using a Zeiss LSM 800 fluorescence microscope (40×) and quantified using ImageJ software.

### Colony formation assays

Colony formation assays were performed as described previously [24]. Briefly, DOX -inducible CHAF1A-knockdown cells were plated at low densities for 48 h in medium containing 1 μg/μl DOX, and treated with the indicated doses of UV or HU. Then, the cells were replaced with fresh medium and incubated for 7–14 days at 37 °C with 5% CO_2_. After washing with PBS, the cultures were fixed with methanol for 20 min and stained with crystal violet overnight. Colonies were imaged using a Chemodoc MP system (Bio-Rad, Hercules, CA, USA) and analyzed using ImageLab (Bio-Rad, Hercules, CA, USA) and ImageJ software. Each experiment was performed in triplicate.

### Local UV radiation

Cells were seeded onto coverslips. Then, pcDNA3.1-Histone H3.1-Flag was transfected to cells and continue to culture for 36 h. Glass coverslips were pre-treated with a solution of 0.1% (w/v) poly-L-lysine for 1 h at 37 °C. These were then rinsed in PBS and cells seeded at the appropriate density the evening before the experiment was due to be performed. Coverslips were rinsed in PBS then individually covered with a piece of Isopore Membrane Filter 3.0 μm (TSTP01300, Millipore), and radiated at 254 nm of UV (100 J/m^2^ dose). The filters were removed and the coverslips returned to the original medium at 37 °C for post-radiation incubation. The method of CPD staining was described in a previous report [28]. Briefly, after post-irradiation incubation, coverslips were rinsed with PBS then washed in CSK buffer (100 mM NaCl, 300 mM sucrose, 10 mM PIPES pH 7.0, 3 mM MgCl_2_) and soaked in CSK buffer plus 0.2% Triton X-100 for 5 min at 25 °C. After two further washes in CSK buffer, cells were fixed in 2% paraformaldehyde for 15 min at 25 °C, and then blocked in 5% BSA (in PBST buffer). Finally, the localization of target protein was analyzed by immunofluorescence. Anti-Cyclobutane Pyrimidine Dimers (CPDs) antibody (Clone TDM-2) was purchased in COSMO BIO USA (Catalog No: CAC-NM-DND-001). CPD indicates the UV damage site.

### Immunofluorescence and HIS-SIM super-resolution microscopy

Cells were fixed with 4.0% paraformaldehyde in PBS for 15 min at room temperature, permeabilized with 0.1% Triton X-100 for 8 min on ice, and then washed three times with PBS. Cells were stained with the indicated primary and secondary HRP-conjugated antibodies after blocked with 5% BSA (in PBS). The coverslips were then mounted onto slides. For super-resolution microscopy, images were taken and reconstructed using a High intelligent and sensitive structured illumination microscope (HIS-SIM) (Guangzhou CSR Biotech Co. Ltd) [29]. HIS-SIM microscopy was used to acquire images to show CHAF1A and RAD18 colocalization. First, the slides were imaged using HIS-SIM with a ×100/1.5 NA oil immersion objective (Olympus). The raw image was exposed for 10 ms and captured with a resolution of 256 × 256 pixels. Then, to improve the resolution and contrast in reconstructed images, sparse deconvolution was used according to a previous report [30]. The images were identically acquired using the IMAGER software (V1.2.4.C) and processed using the Image J software.

### Recombinant protein expression and purification

Recombinant GST-tagged or His-tagged CHAF1A, RAD18, PCNA, RPA1, RPA2, RPA3 and CHAF1A truncated forms were transformed into *Escherichia coli* BL21 (DE3). Then, 100 mL of LB medium with ampicillin was seeded with 1 mL of DE3 cells transformed with the indicated plasmid and grown at 37 °C until the OD600 was reached to 1-1.2. The mixture was then transferred to 25 °C and induced by the addition of 0.5 mM isopropyl-β-D-thiogalactoside (IPTG) for 5 h. Bacteria were collected by centrifugation at 5000 g for 5 min. The bacteria then suspended in 10 mL of GST extraction buffer (4.3 mM Na_2_HPO4, 1.47 mM KH_2_PO4, 137 mM NaCl, 0.1% Triton X-100, pH 7.3) or His lysis buffer (50 mM sodium phosphate, pH 8.0, 300 mM NaCl, and 10 mM imidazole) followed by incubation with GST resin (70541-3; Millipore, Billerica, MA, USA) or Ni-NTA resin (70666-3; Millipore) at 4 °C for 3 h. The GST-resin and His-resin were then washed four times using GST lysis buffer and His wash buffer (50 mM sodium phosphate, pH 8.0, 300 mM NaCl, and 20 mM imidazole), respectively. To obtain GST-tag or His-tag fused protein, the resin was eluted using GST (50 mM Tris-HCl, and 50 mM reduced form of glutathione, pH 8.0) or His elution buffer (50 mM sodium phosphate, pH 8.0, 300 mM NaCl, and 250 mM imidazole).

### GST/His pull-down assays

The GST pull-down assay was performed as described previously [31]. Briefly, the GST beads coupled to GST-fusion proteins were equilibrated with cell lysis buffer (50 mM Tris-HCl pH 7.5, 150 mM NaCl, and 0.5% NP-40) and then incubated with appropriate amount of cell lysates for indicated time at 4 °C. GST pull-down samples were collected and washed four times with cell lysis buffer. For His pull-down assay, the Ni-NTA beads coupled to His-fusion proteins were equilibrated with 1×PBS buffer and then incubated with appropriate amount of recombinant GST-tag protein for indicated time at 4 °C. His pull-down samples were collected and washed four times with 1×PBS buffer. After SDS-PAGE, the GST/His pull-down samples were analyzed by Coomassie bright blue (CBB) or western blotting.

### ssDNA/dsDNA pull-down assays (streptavidin pull-down assays)

The ssDNA/dsDNA pull-down assay was modified based on previous study [32].To generate ssDNA/dsDNA, biotinylated DNA oligomers (5’-AAC CTG TCG TGC CAG CTG CA-biotin-3’) were first annealed to complementary ssDNA (5’-TGC AGC TGG CAC GAC AGG TTT TAA TGA ATC GGC CAA CGC GCG GGG AGA GGC GGT TTG CGT ATT GGG CGC TCT TCC GCT TCG CAG CGA GTC-3’) with molar ratio 1:4. The annealed ssDNA/dsDNA product (100 pmol) was first bound with Streptavidin Magnetic Bead (11641786001, Roche), followed by washing with binding buffer (10 mM Tris-HCl [pH 7.5], 100 mM NaCl, 10% glycerol, 0.01% NP-40, and BSA 10 mg/mL) and then coated with recombinant CHAF1A-His (WT or mutants) for 1 h. Alternatively, The annealed ssDNA/dsDNA product (100 pmol) was first bound with Streptavidin Magnetic Bead, followed by washing with binding buffer and then coated with recombinant RPA complex (RPA1-His, RPA2-His and RPA3-His) for 1 h and then coated with recombinant CHAF1A-His for 1 h. Then, the beads carrying RPA-CHAF1A-ssDNA/dsDNA were incubated in recombinant RAD18-GST for 30 min, followed by extensive washing with NETN buffer (20 mM Tris-HCl [pH 7.5], 500 mM NaCl, 0.5% NP-40, 5 mM EDTA) three times. The proteins bound to beads were then subjected to western blotting.

### In vitro ubiquitination assay

The in vitro ubiquitination assay was modified based on previous study [33]. Prior to this assay, the proteins required for the reaction need to be purified. The purification of GST/His tagged proteins is detailed above. HA-labeled RAD6A and PCNA were purified from HEK293T cells. Briefly, pcDNA3.1-RADA6-HA and pCMV-PCNA-HA vectors were transfected into HEK293T cells. After 48 h, the total protein cell lysate was enriched with CoIP assay using anti-HA antibodies. Finally, the proteins were eluted competitively from the bead with HA peptide (HY-P0239, MCE). First, His tagged UBE1 protein (23-021-M, sigma) (100 ng) was pre-incubated with ubiquitin (U5507, sigma) (1 μg), ATP (1 mM) and MgCl_2_ (1 mM) at 25 °C in reaction buffer (25 mM Tris pH 8.0, 150 mM NaCl, 2 mM ZnCl_2_ and 5 mM β-ME) for 5 min. Subsequently, the indicated amount of His tagged CHAF1A is added. Then, HA tagged RAD6A protein (100 ng) and GST tagged RAD18 (200 ng) and HA tagged PCNA (500 ng) were added and the reaction (The total volume is 20 μL) incubated at 30 °C for 2 h. Finally, samples were boiled in SDS loading buffer and were then subjected to western blotting.

### Quantification and statistical analysis

All statistical analyses were performed using Prism (version 8.0.2, GraphPad). Significant differences were determined using *P* values (^*^P < 0.05, ^**^P < 0.01). If the data conformed to a normal distribution, unpaired t tests were used, or a one-way analysis of variance (ANOVA) when comparing more than two samples. All plotted values represent the mean ± standard deviation (SD). Data in graphs displaying fold changes represent the mean ± SD of fold changes calculated from the mean of control samples. ImageJ software was used for gray value analysis.

## Results

### CHAF1A enhances PCNA K164 monoubiquitination in response to DNA replication stress in cancer cells

**Fig. 1:**
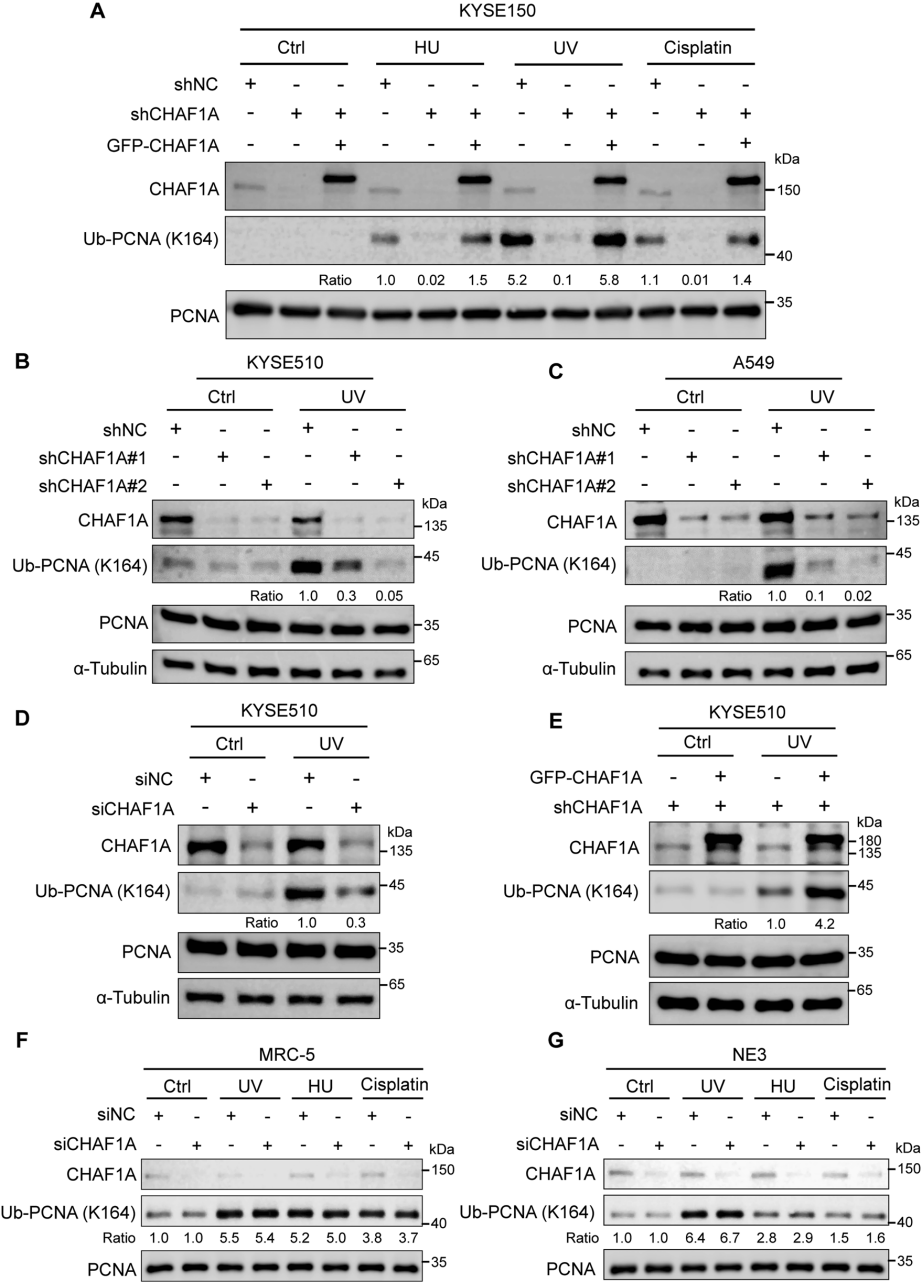
CHAF1A promotes PCNA K164 monoubiquitylation induced by UV, HU or cisplatin. **A** CHAF1A-knockdown KYSE150 cells were transfected with plasmid encoding GFP-CHAF1A and then treated with HU (2 mM 4 h), UV (100 J/m^2^ 1 h) or cisplatin (30 μM 6 h). Whole-cell lysates were analyzed using western blotting with the indicated antibodies. **B** CHAF1A-knockdown KYSE510 cells were treated with or without 100 J/m^2^ UV for 1 h. Whole-cell lysates were analyzed using western blotting with the indicated antibodies. **C** CHAF1A-knockdown A549 cells were treated with or without 100 J/m^2^ UV for 1 h. Whole-cell lysates were analyzed using western blotting with the indicated antibodies. **D** KYSE510 cells were transfected with control or CHAF1A siRNA oligos for 24 h and then treated with or without 100 J/m^2^ UV for 1 h. Whole-cell lysates were analyzed using western blotting with the indicated antibodies. **E** CHAF1A-knockdown KYSE510 cells were transfected with CHAF1A-GFP plasmid and treated with or without 100 J/m^2^ UV for 1 h. (**F** and **G**) MRC-5 cells or NE3 cells were transfected with control or CHAF1A siRNA oligos for 24 h and then treated with UV (100 J/m^2^ 1 h), HU (2 mM 4 h) or cisplatin (30 μM 6 h). Whole-cell lysates were analyzed using western blotting with the indicated antibodies.

Previous studies have reported colocalization of CHAF1A and PCNA in response to UV, suggesting a close relationship between CHAF1A and PCNA under UV radiation [34]. Therefore, we speculated that CHAF1A may influence UV-induced monoubiquitination of PCNA at K164, which is a key step in the TLS pathway. After HU, UV and cisplatin treatment, PCNA K164 monoubiquitination level was significantly increased in KYSE150 cells, which was inhibited by CHAF1A knockdown and rescued by restoring CHAF1A expression **(**Fig. 1A**)**. Then, KYSE510 and A549 cell lines were exposed to UV radiation to activate PCNA K164 monoubiquitination, and western blotting analysis showed that CHAF1A deficiency significantly inhibited the level of PCNA K164 monoubiquitination (Fig. 1B, C**)**. We used siRNA to knock down CHAF1A and detect UV-induced PCNA K164 monoubiquitination, and found that PCNA K164 monoubiquitination was inhibited by CHAF1A knockdown in KYSE510 cells **(**Fig. 1D**)**. Moreover, CHAF1A overexpression significantly promoted UV-induced PCNA K164 monoubiquitination **(**Fig. 1E**)**. In addition, in lung normal cell line MRC-5 and esophageal normal cell line NE3, knockdown CHAF1A had no significant effect on UV-induced PCNA K164 monoubiquitination (Fig. 1F, G**)**. Interestingly, CHAF1B knockdown did not affect UV-induced PCNA K164 monoubiquitination **(**Supplementary Figure 1A and 1B**)**. We examined the effects of CHAF1A on HU-induced PCNA K164 mono-ubiquitination. Western blotting analysis showed that CHAF1A knockdown significantly inhibited PCNA K164 mono-ubiquitination induced by HU in KYSE510 cells and A549 cells **(**Supplementary Figure 2A and 2B**)**. These results strongly suggest that CHAF1A promotes PNCA K164 monoubiquitination in response to DNA replication stress in cancer cells.

### CHAF1A promotes the TLS pathway and inhibits DNA damage under DNA replication stress

**Fig. 2:**
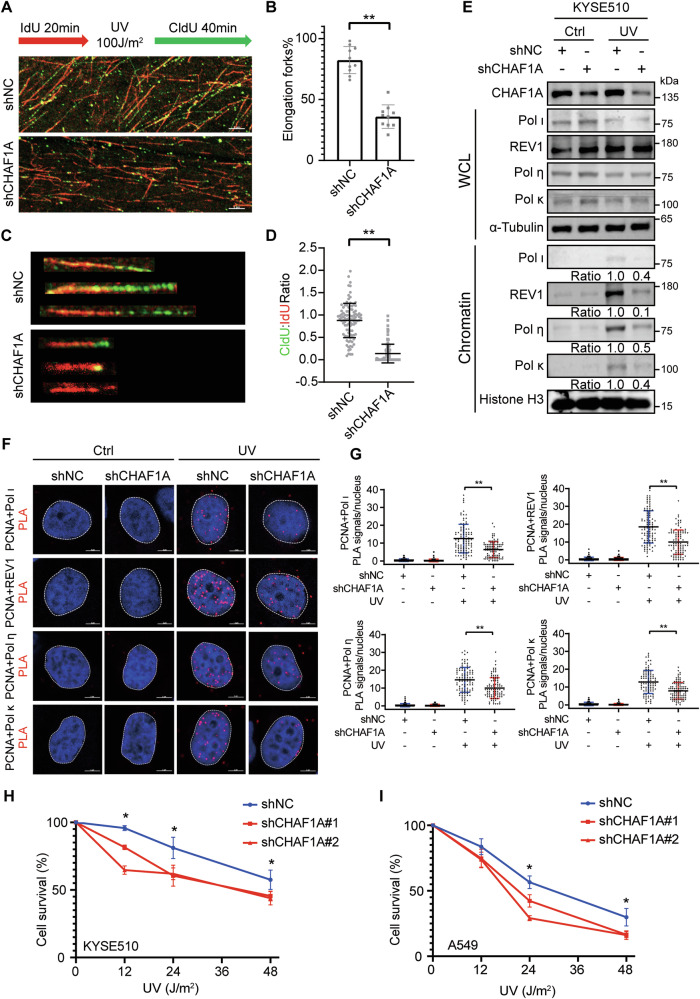
CHAF1A is required for the TLS pathway. **A** DNA fiber assay was used to measure DNA replication in inducible CHAF1A knockdown KYSE510 cells. Schematic of alternative IdU/CldU pulse-labeling protocol to evaluate fork status. Representative images of DNA fiber assays are shown. **B** Image J was used to calculate the percentage of elongation forks in each field of view (n = 10). Data represent means ± SEM from three independent experiments. ^**^P < 0.01; t-test. **C** IdU and CldU staining in a single DNA fiber were presented as representative images. Data are plotted in **D** (*n* = 100). Data are representative of at least three independent experiments. ^**^P < 0.01; t-test. **E** CHAF1A-knockdown KYSE510 cells were treated with or without 100 J/m^2^ UV for 1 h. Cell fractionation was performed to detect indicated antibodies. **F** CHAF1A-knockdown KYSE510 cells were treated with or without 100 J/m^2^ UV for 1 h. PLA assays was used to detect the interaction between PCNA and TLS polymerases (Pol ι, REV1, Pol η, and Pol κ) (scale bar, 5 μm), and (**G**) each spot represents the number of PLA foci in an individual nucleus (n = 100). Data represent means ± SEM from three independent experiments. ^**^P < 0.01; one-way ANOVA. **H** and **I** Colony formation assay for KYSE510 cells or A549 cells with treatment of the indicated dose of UV (*n* = 3). Data represent the mean ± SEM from three independent experiments. ^*^P < 0.05; one-way ANOVA.

Since CHAF1A positively regulated PCNA monoubiquitination, we speculated that CHAF1A may also influence the TLS pathway. Activation of the TLS pathway ensures the restart and elongation of the stalled replication forks. Therefore, to investigate the contribution of CHAF1A deficiency to UV-damaged DNA replication, we monitored replication fork progression on a single DNA fiber. CHAF1A-knockdown KYSE510 cells were labeled with 5-iodo-2-deoxyuridine (IdU) pulses for 20 min, then radiated with UV, and then labeled with 5-chloro-2-deoxyuridine (CldU) for 40 min. The CHAF1A knockdown reduces the percentage of elongation replication forks (Fig. 2A, B**)**, suggesting that CHAF1A facilitates the restarting of stalled replication forks. In addition, the ratio of CldU to IdU staining length was measured to determine the effect of CHAF1A on the rate of DNA replication after UV damage. The results showed that CHAF1A knockdown inhibited the DNA replication rate after UV radiation (Fig. 2C, D**)**.

Then, we used DNA fiber assays to directly examine the impact of CHAF1A on DNA replication forks under HU treatment. Nascent DNA was sequentially labeled with CldU and IdU with low concentrations of HU (50 μM). The ratio of IdU- to CldU-labeled replication tracts were lower in shCHAF1A cells than in shNC cells **(**Supplementary Figure 3A and 3B**)**. To investigate whether CHAF1A knockdown affects replication fork restarting after HU treatment, we also performed DNA fiber assay. As reported previously [35], IdU detection after CldU labeling indicated the restart of the replication fork after HU treatment. However, the CHAF1A knockdown significantly reduced replication fork restart after HU treatment **(**Supplementary Figure 3C and 3D**)**, suggesting that CHAF1A facilitates the restarting of stalled replication forks under HU treatment.

PCNA K164 monoubiquitination causes the replacement of replicative DNA polymerase with TLS polymerase [36]. In humans, at least four Y-family TLS polymerases can be recruited to sites of arrested replication, including Pol ι, REV1, Pol η and Pol κ [37]. Therefore, we examined UV-induced recruitment of Y-family TLS polymerase to further explore whether CHAF1A affects the TLS pathway. Cell fractionation assays showed that CHAF1A knockdown inhibited the recruitment of multiple TLS polymerases in chromatin after UV radiation **(**Fig. 2E**)**. PLA assays showed that UV enhances the interaction between Y-family TLS polymerase and PCNA, which was inhibited by CHAF1A knockdown (Fig. 2F, G**)**. We also used PLA assay to verify the effect of CHAF1A on the interaction between PCNA and Polη under HU treatment. The interaction between PCNA and Polη was enhanced after HU treatment and was reduced in shCHAF1A cells **(**Supplementary Figure 3E and 3F**)**. Moreover, in order to verify the effect of CHAF1A on the survival ability of cancer cells under DNA replication stress, we treated KYSE510 and A549 cells with different doses of UV and measured cell survival with a colony formation assay. The results showed that shCHAF1A cells were more sensitive to UV than shNC cells (Fig. 2H, I**)**. Similarly, shCHAF1A cells were more sensitive to HU than shNC cells **(**Supplementary Figure 3G and 3H**)**. These results indicated that the promotion of the TLS pathway by CHAF1A is necessary for the survival of cancer cells under DNA replication stress.

SsDNA gaps formed by DNA replication stress activate the RPA/ATR/ CHK1 pathway in human cells [19]. We found that CHAF1A did not affect the phosphorylation of RPA2 and CHK1, which are hallmarks of ATR pathway activation **(**Supplementary Figure 4A**)**. Continued replication fork stalling may lead to DNA damage and cell death [19]. To understand the effect of CHAF1A on DNA damage level after DNA replication stress, we examined the levels of the DNA damage marker γH2AX, and found that CHAF1A knockdown significantly increased DNA damage accumulation (γH2AX intensity) after UV or HU treatment, which was rescued by restoring CHAF1A expression (GFP-CHAF1A positive) **(**Supplementary Figure 4B**)**. These results suggest that CHAF1A attenuates DNA damage following DNA replication stress.

### CHAF1A promotes PCNA K164 monoubiquitination independent of CHAF1A-PCNA interaction

**Fig. 3:**
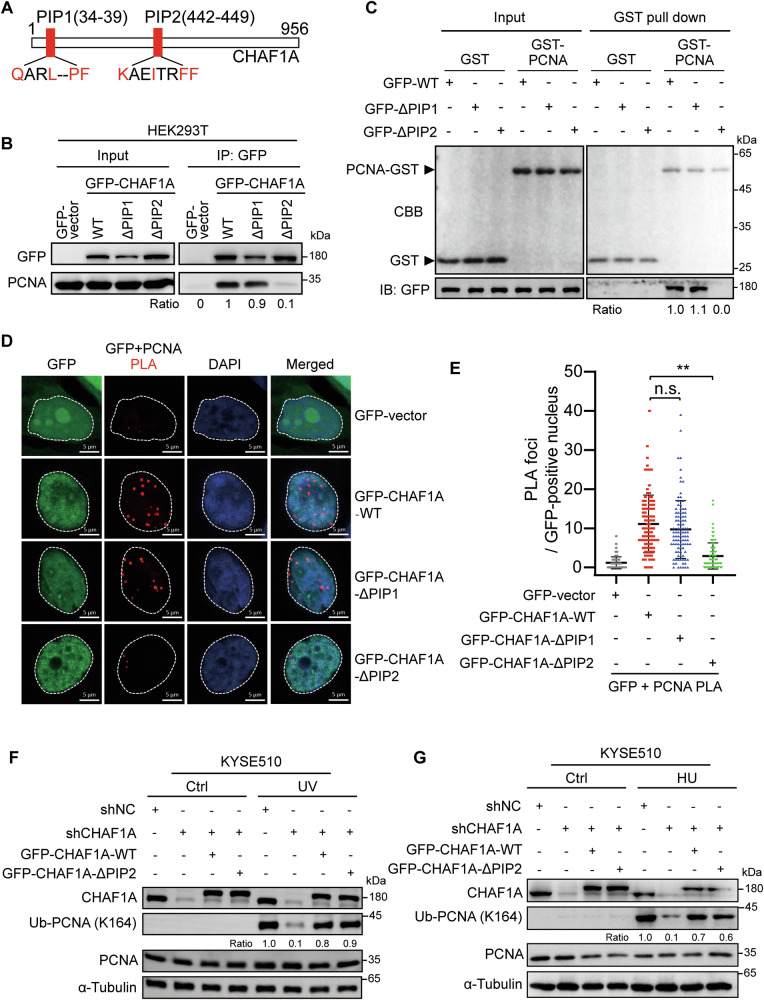
The CHAF1A PIP2 domain mediates the CHAF1A-PCNA interaction, but is not necessary for PCNA monoubiquitination. **A** Schematics of CHAF1A PIP domains. **B** Interactions of GFP-CHAF1A (WT, ΔPIP1, and ΔPIP2) with PCNA in HEK293T cells assessed by CoIP using anti-GFP antibody. ΔPIP1: PIP1 domain deletion, ΔPIP2: PIP2 domain deletion. **C** The binding of CHAF1A mutants to PCNA was detected by in vitro GST pull-down assay. GST-PCNA beads were incubated with lysates of HEK293T expressing GFP-CHAF1A-WT, GFP-CHAF1A-ΔPIP1 or GFP-CHAF1A-ΔPIP2. **D** The in-situ interaction of GFP-CHAF1A (WT, ΔPIP1, and ΔPIP2) with PCNA in KYSE510 cells was evaluated by PLA assay using GFP and PCNA antibodies. GFP-v was used as a negative control (scale bar, 5 μm). **E** The number of PLA foci in GFP-positive nuclei was counted, each spot represents the number of PLA foci in an individual nucleus (*n* = 100). Data are representative of at least three independent experiments. n.s., *P* > 0.05, ^**^P < 0.01; one-way ANOVA. **F** CHAF1A-knockdown KYSE510 cells were transfected with GFP-CHAF1A-WT or GFP-CHAF1A-ΔPIP2 vectors, and then treated with or without 100 J/m^2^ UV for 1 h. Whole-cell lysates were analyzed using western blotting with the indicated antibodies. **G** CHAF1A-knockdown KYSE510 cells were transfected with GFP-CHAF1A-WT or GFP-CHAF1A-ΔPIP2 vectors, and then treated with or without 2 mM HU for 4 h. Whole-cell lysates were analyzed using western blotting with the indicated antibodies.

Previous studies have reported that CHAF1A contains two PCNA-interaction peptides (PIP1 and PIP2) **(**Fig. 3A**)** [38]. However, the domain on which CHAF1A interacts with PCNA is still controversial. Ben-Shahar et al. found that CHAF1A interacts with PCNA through its PIP1 domain [38], while Cheng et al. demonstrated that CHAF1A interacts with PCNA dependent on the PIP2 domain [39]. To confirm which PIP domain of CHAF1A affected the interaction with PCNA, we performed CoIP assays with anti-GFP antibody in HEK293T cells transfected with GFP-vector, GFP-CHAF1A-WT, GFP-CHAF1A-ΔPIP1, or GFP-CHAF1A-ΔPIP2. Western blotting analysis showed that CHAF1A interacts with PCNA dependent on the PIP2 domain **(**Fig. 3B**)**. In vitro GST pull-down assay also proves that PIP2 domain is necessary for CHAF1A to interact with PCNA **(**Fig. 3C**)**. Then, we introduced GFP-vector, GFP-CHAF1A-WT, GFP-CHAF1A-ΔPIP1, or GFP-CHAF1A-ΔPIP2 into KYSE510 cells and performed PLA assay using anti-GFP and anti-PCNA antibodies. The PLA assay demonstrated that the absence of CHAF1A PIP2 domain significantly inhibited the interaction between CHAF1A and PCNA **(**Fig. 3D, E**)**.

Next, we examined whether PCNA K164 mono-ubiquitination regulated by CHAF1A is dependent on the interaction between CHAF1A and PCNA. We restored the expression of CHAF1A-WT and CHAF1A-ΔPIP2 mutants in the shCHAF1A cells. We detected PCNA K164 monoubiquitination after UV radiation or HU treatment. Western blotting analysis showed that CHAF1A knockdown significantly reduced PCNA K164 monoubiquitination, while overexpression of both GFP-CHAF1A-WT and GFP-CHAF1A-ΔPIP2 significantly restored PCNA K164 monoubiquitination **(**Figs. 3F and G**)**. Together, these results suggest that the interaction of CHAF1A with PCNA requires the PIP2 domain. Meanwhile, the enhancement of PCNA monoubiquitination by CHAF1A was independent of the interaction between CHAF1A and PCNA.

### CHAF1A stimulates PCNA monoubiquitination in a RAD18-dependent manner

**Fig. 4:**
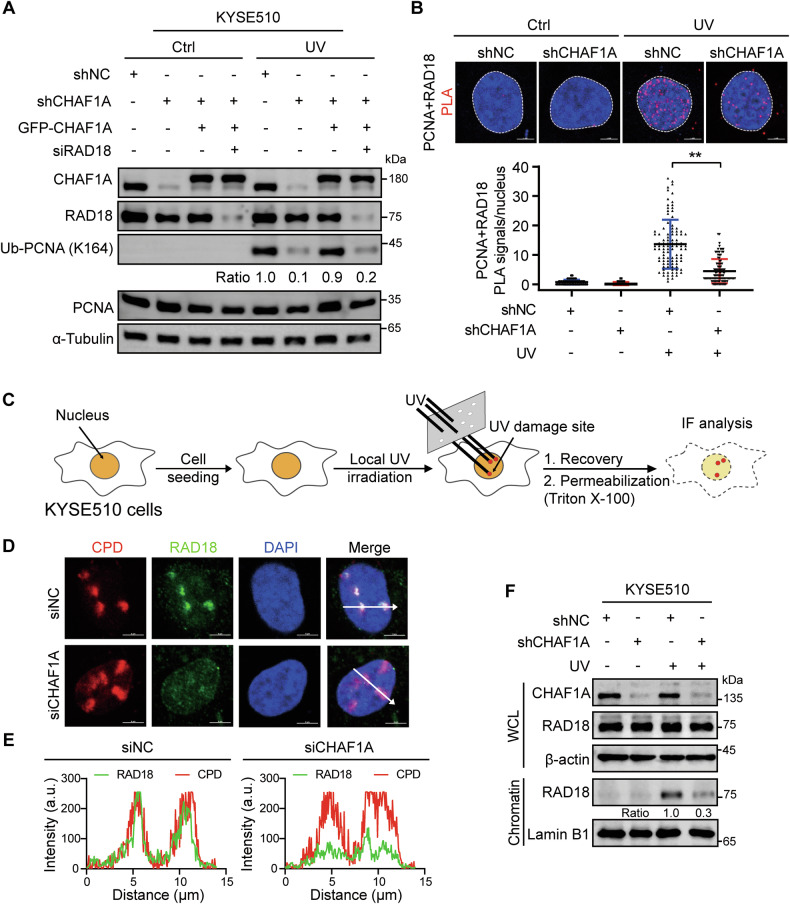
CHAF1A promotes RAD18 recruitment after UV damage. **A** CHAF1A-knockdown KYSE510 cells were transfected with indicated siRNAs before transfection with plasmid encoding GFP-CHAF1A and then treated with or without 100 J/m^2^ UV for 1 h. **B** CHAF1A-knockdown KYSE510 cells were treated with or without 100 J/m^2^ UV for 1 h. PLA assay was used to detect the interaction between RAD18 and PCNA (scale bar, 2 μm), and each spot represents the number of PLA foci in an individual nucleus (*n* = 100). Data are representative of at least three independent experiments. ^**^P < 0.01; one-way ANOVA. **C** Experimental scheme for UV damage induction in spots. To analyze the behavior of RAD18 after UV damage, we selected and micro-radiated with 100 J/m^2^ UV in KYSE510 cells. **D** After UV damage, the cells underwent immunofluorescence (IF) analysis. (**E)** ZEN software was used to calculate the fluorescence intensity on the white arrow in (D). **F** CHAF1A-knockdown KYSE510 cells were treated with or without 100 J/m^2^ UV for 1 h. Cell fractionation was performed to detect indicated antibodies.

Since the interaction between CHAF1A and PCNA is not required for PCNA K164 monoubiquitination, how does CHAF1A affect the molecular mechanism of PCNA K164 monoubiquitination? RAD18 is an E3 ubiquitin ligase that directly mediates the monoubiquitination of PCNA at K164 during TLS [40]. Here, we hypothesized that CHAF1A promotes PCNA K164 monoubiquitination mediated by RAD18. To test this hypothesis, KYSE510 cells transfected with GFP-CHAF1A (WT) were exposed to UV radiation or HU, and western blotting analysis showed that CHAF1A overexpression significantly increased PCNA monoubiquitination, which was inhibited by RAD18 knockdown **(**Fig. 4A, Supplementary Figure 5H**)**. This result indicated that the promotion of PCNA monoubiquitination by CHAF1A is dependent on RAD18.

Next, we hypothesized that CHAF1A affected the interaction between RAD18 and PCNA, which in turn affected PCNA K164 monoubiquitination. To test this conjecture, we performed PLA assays to detect the effect of CHAF1A on the interaction between PCNA and RAD18. We found that the interaction between PCNA and RAD18 was significantly enhanced by UV radiation, and was reduced in CHAF1A-knockdown cells **(**Fig. 4B**)**. Then, we tested the effect of CHAF1A knockdown on UV-induced RAD18 chromatin binding by cell fractionation assays, and found that CHAF1A knockdown significantly inhibited RAD18 binding on chromatin (Fig. 4F**)**.

To investigate repair site histone dynamics that allowed us to visualize UV-damage site recruitment of the histone variant H3.1, we employed an assay of site-specific cell damage based on transient transfection with epitope-labeled histones (Flag-H3.1), which can be easily distinguished from endogenous histones [41] **(**Supplementary Figure 5A**)**. UV-induced CPD can be detected by anti-CPD antibodies [42]. After local UV radiation, we monitored local recruitment of Flag-H3.1 at UV damage sites. Consistent with previous studies, either the CHAF1A knockdown or the CHAF1B knockdown prevented Flag-H3.1 recruitment at the UV damage site **(**Supplementary Figure 5B, 5C, 5D and 5E**)**. Next, we observed RAD18 recruitment at UV damage sites **(**Fig. 4C**)**, and the results showed that CHAF1A knockdown significantly inhibited RAD18 recruitment at UV damage sites (Fig. 4D, E**)**. However, the CHAF1B knockdown does not affect RAD18 recruitment at UV damage sites **(**Supplementary Figure 5F and 5G**)**. Together, these results demonstrate that CHAF1A promotes PCNA K164 monoubiquitination mediated by RAD18 and independent of its histone assembly function.

### CHAF1A directly interacts with RAD18 through its C-terminal domain

**Fig. 5:**
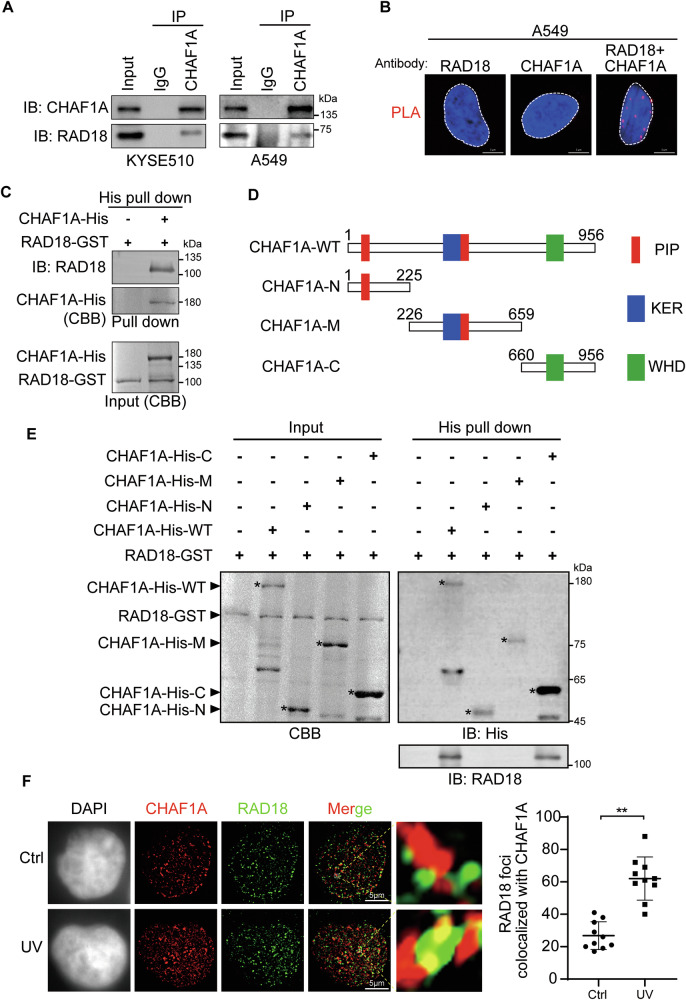
CHAF1A directly interacts with RAD18. **A** Association of endogenous CHAF1A with RAD18 in KYSE510 and A549 cells assessed by CoIP using anti-CHAF1A antibodies. **B** PLA assay for the interaction of CHAF1A and RAD18 in A549 cells (scale bar, 5 μm). **C** CHAF1A was fused to His (CHAF1A-His) and purified for His pull-down assays with GST-fused RAD18 (RAD18-GST). (**D**) Schematic of CHAF1A mutants. **E** The N-terminal (N), Middle (M) or C-terminal domain (**C**) of CHAF1A were fused to His and purified for His pull-down assays with GST-fused RAD18 (RAD18-GST). CBB: Coomassie brilliant blue. **F** KYSE510 cells were treated with or without 100 J/m^2^ UV for 1 h. Fluorescence microscopy was used to detect CHAF1A and RAD18 (scale bar, 5 μm), and each spot represents the number of RAD18 foci co-located with CHAF1A in an individual nucleus (*n* = 10). Data represent means ± SEM from three independent experiments. ^**^P < 0.01; t-test.

Since CHAF1A is required for RAD18’s recruitment, we hypothesize that CHAF1A is physically connected to RAD18. To verify the interaction between CHAF1A and RAD18, we performed CoIP assay in KYSE510 cells and A549 cells, and found that exogenous CHAF1A interacted with endogenous RAD18 **(**Fig. 5A**)**. The interaction between CHAF1A and RAD18 was also confirmed by PLA assay **(**Fig. 5B**)**. Moreover, to examine whether CHAF1A and RAD18 interact directly, we expressed His-fused CHAF1A and GST-fused RAD18 in *Escherichia coli* and purified them for His pull down assay. The results showed that RAD18-GST was pulled down by CHAF1A-His, indicating that CHAF1A interacted directly with RAD18 **(**Fig. 5C**)**. To further elucidate the interaction between CHAF1A and RAD18, the His fusion proteins containing different CHAF1A domains, including the N-terminal segment, middle segment, and C-terminal segment, were purified for His-pull down assays with RAD18-GST in vitro **(**Fig. 5D**)**. Similar to the-full length CHAF1A, the individual C-terminal domain of CHAF1A interacted with RAD18 **(**Fig. 5E**)**. Then, we used super-resolution microscopy HIS-SIM to observe the colocalization of CHAF1A with RAD18 after UV treatment. After UV treatment, the co-localization of CHAF1A and RAD18 was significantly increased **(**Fig. 5F**)**. Together, these results suggest that CHAF1A promotes PCNA K164 mono-ubiquitination in a RAD18-dependent manner, and in addition, CHAF1A directly interacts with RAD18 through its C-terminal domain.

### The CHAF1A recruits RAD18 to the stalled replication fork, enhancing PCNA K164 monoubiquitination

**Fig. 6:**
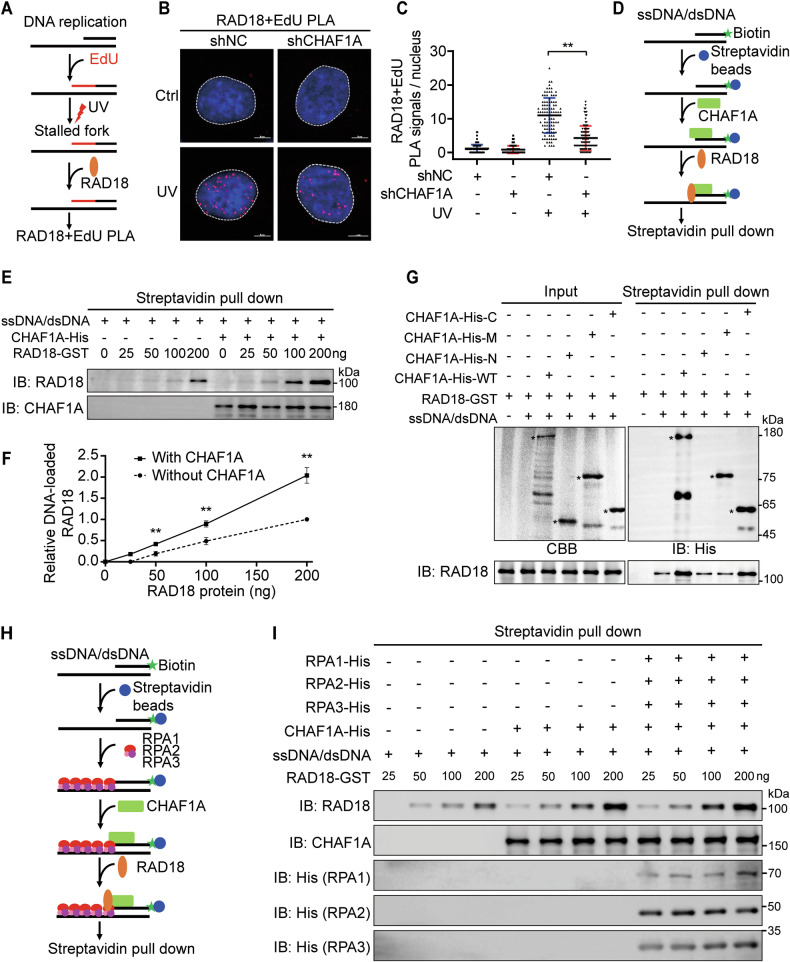
CHAF1A recruits RAD18 to stalled replication forks. **A** Schematic of RAD18-EdU PLA assay. **B** CHAF1A-knockdown KYSE510 cells were treated with or without 100 J/m^2^ for 1 h. Biotin was connected to EdU by a Click-iT reaction, and then PLA assay was performed with anti-RAD18 and anti-biotin antibodies. **C** Statistical diagram of PLA foci for **C**, *n* = 100. Data are representative of at least three independent experiments. ^**^P < 0.01; one-way ANOVA. **D** Schematic of ssDNA/dsDNA pull-down assay. **E** Biotinylated ssDNA/dsDNA were first bound with streptavidin beads and coated with bacterially produced recombinant CHAF1A, followed by incubating with the indicated concentration of recombinant RAD18. The relative DNA-loaded of RAD18 is calculated by the gray value of RAD18 in **F**. Data represent means ± SEM from three independent experiments. ^**^P < 0.01; one-way ANOVA. **G** Biotinylated ssDNA/dsDNA were first bound with streptavidin beads and coated with bacterially produced recombinant wild-type and CHAF1A mutants, followed by incubating with recombinant RAD18, followed by immunoblotting with RAD18 antibody. **H** Schematic of ssDNA/dsDNA pull-down assay with RPA complex. **I** Biotinylated ssDNA/dsDNA were first bound with streptavidin beads and coated with bacterially produced recombinant RPA complex (RPA1-His, RPA2-His and RPA3-His), followed by incubation with recombinant CHAF1A and then with recombinant RAD18 at the indicated concentrations. The relative DNA-loaded of RAD18 is calculated by immunoblotting with RAD18 antibody.

We were intrigued to see that CHAF1A is necessary for RAD18 recruitment to PCNA in response to DNA damage, and so next investigated whether the knockdown of CHAF1A affects RAD18 recruitment to stalled replication forks. To further verify the association of RAD18 with stalled replication forks, we carried out a PLA assay. We treated with EdU combined with UV to label the stalled replication forks, then connected biotin to EdU with a Click-iT reaction, and finally performed PLA assay with anti-biotin and anti-RAD18 antibodies **(**Fig. 6A**)**. As shown in Fig. 6B, C, RAD18/EdU PLA foci is almost undetectable without UV. Cells treated with UV exhibited a significant increase in the number of RAD18/EdU PLA foci. The number of RAD18/EdU foci increased by UV was significantly reduced in shCHAF1A cells **(**Fig. 6B, C**)**.

Next, we explored how CHAF1A affects RAD18 recruitment in stalled replication forks. CHAF1A contains two DNA-binding domains, KER and WHD, that mediate CHAF1A to bind DNA directly **(**Fig. 5D**)**. We conjectured that CHAF1A accelerates RAD18 binding on the stalled replication fork, thereby facilitating the TLS pathway. SsDNA/dsDNA structure is a special structure on the stalled replication fork during DNA replication stress [32, 43]. To test this hypothesis, we introduced a biotin-labelled ssDNA/dsDNA oligonucleotides to mimic the structure of stalled replication forks **(**Supplementary Figure 6A**)**. The streptavidin pull-down assays showed that biotin-ssDNA/dsDNA was able to efficiently pull down His-tagged CHAF1A **(**Supplementary Figure 6B**)**. Previous studies suggested that RAD18 could be bound to the stalled replication forks via its SAP domain [33]. In this study, we found that GST-tagged RAD18 possess weak DNA binding ability **(**Supplementary Figure 6C**)**. Interestingly, CHAF1A-coated biotin-ssDNA/dsDNA can significantly enhance RAD18 binding to ssDNA/dsDNA **(**Fig. 6D, E, and F**)**. To further demonstrate which domain of CHAF1A mediates RAD18 binding to ssDNA/dsDNA, the His fusion proteins containing different CHAF1A domains, including the N-terminal segment, middle segment, and C-terminal segment, were purified for streptavidin pull-down assays with RAD18-GST in vitro. Like the full-length CHAF1A, the middle segment and C-terminal segment of CHAF1A containing the DNA-binding domains KER and WHD, respectively, can bind ssDNA/dsDNA. In contrast to the middle segment of CHAF1A, the C-terminal region of CHAF1A containing the RAD18 binding domain can significantly promotes RAD18 binding to ssDNA/dsDNA **(**Fig. 6G**)**.

The coating of the ssDNA binding protein RPA complex on stalled replication fork is an early step in the response to DNA replication stress. To explore the effect of the RPA complex on the binding of CHAF1A and RAD18 on stalled replication fork, we repeated the ssDNA/dsDNA binding assay of Fig. 6D in the presence of RPA (RPA1, RPA2 and RPA3) **(**Fig. 6H**)**. We found that the recombinant RPA can bind to ssDNA/dsDNA, and did not affect the binding of CHAF1A to ssDNA/dsDNA. The RPA complex had also no significant effect on CHAF1A-mediated RAD18 binding on ssDNA/dsDNA **(**Fig. 6I**)**.

**Fig. 7:**
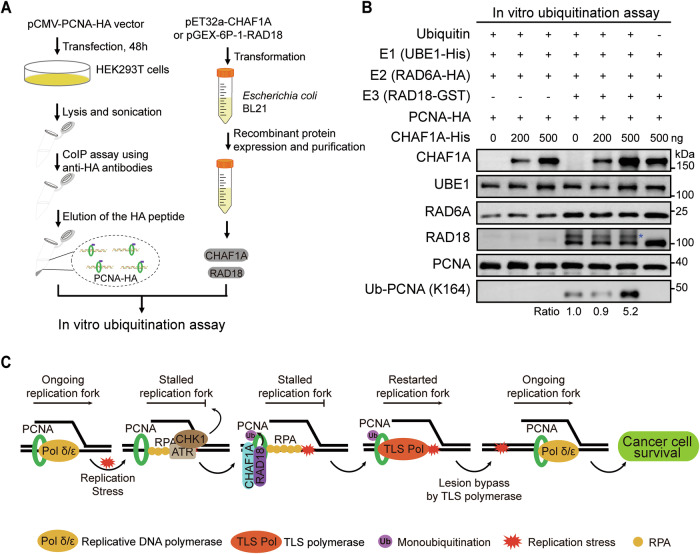
CHAF1A promotes RAD18/RAD6A-mediated PCNA K164 monoubiquitination in vitro. **A** Schematic of in vitro ubiquitination assay. **B** In vitro ubiquitination assay of PCNA. The blue asterisk indicates the ubiquitinated RAD18. **C** The working model of CHAF1A in regulating TLS pathway. Replicative DNA polymerases Pol δ/ε dissociate from the replication fork when the ongoing replication fork encounters replication stress. Then, the ATR-dependent RPA-CHK1 signaling pathway is activated. CHAF1A recruits RAD18 to the stalled replication fork in a DNA binding-dependent manner. RAD18 accelerates PCNA K164 mono-ubiquitination on the stalled replication fork. PCNA K164 mono-ubiquitination promotes the conversion of replicative DNA polymerase Polδ/ε into TLS DNA polymerases (REV1, Pol η, Pol κ and Pol ι). The TLS pathway activated by CHAF1A enhances replication fork restart and damage-tolerant DNA replication, which in turn leads to cancer cell survival under DNA damage.

Then, in vitro ubiquitination assay was carried out using in vitro translated/immuno-purified PCNA and purified recombinant proteins **(**Fig. 7A**)**. Recombinant RAD18 can induce ubiquitination at PCNA K164 site in vitro. When the recombinant CHAF1A reaches a certain concentration, the ubiquitination level of PCNA K164 is significantly enhanced **(**Fig. 7B**)**. Taken together, these results strongly suggest that CHAF1A mediates the recruitment of RAD18 to stalled replication forks, which in turn promotes PCNA monoubiquitination.

## Discussion

Endogenous and exogenous DNA damage can damage rapidly proliferating cancer cells; however, these cells can bypass DNA damage to maintain genomic stability and survive via the TLS pathway mediated by PCNA K164 monoubiquitination. CHAF1A traditionally functions as a PCNA partner for chromatin remodeling functions and is involved in histone assembly along nascent DNA strands. In our study, we identified a new function of CHAF1A that plays an important regulatory role in the TLS pathway **(**Fig. 7C**)**. Specifically, Replicative DNA polymerases Pol δ/ε dissociate from the replication fork when the ongoing replication fork encounters replication stress. Then, the ATR-dependent RPA-CHK1 signaling pathway is activated. CHAF1A promotes the recruitment of RAD18 on the stalled replication fork to enhance PCNA K164 monoubiquitination. Once PCNA is monoubiquitinated at K164, the Y-family DNA polymerases replace Pol δ/ε to allow stalled replication forks to proceed. High expression of CHAF1A enables cancer cells to enhance the TLS pathway in response to endogenous and exogenous DNA damage, improve the survival ability of cancer cells, and then promote cancer progression. Interestingly, this study found that CHAF1A knockdown did not affect PCNA K164 monoubiquitination in normal cells. This result suggests that CHAF1A regulation of the TLS pathway appears to be cancer cell specific.

CHAF1A is a subunit of the CAF-1 complex, which is recruited to the DNA replication and DNA damage sites by PCNA and is responsible for histone assembly and chromatin remodeling [44]. During DNA replication stress, PCNA K164 monoubiquitination rapidly initiates the TLS pathway, which recruits lesion-tolerant Y-family polymerases to stalled replication sites [17]. Dungrawala et al. found that most PCNA and CAF-1 molecules were dissociated from stalled forks under replication stress, but PCNA and CAF-1 were present on stalled forks during a long period of DNA replication stress [45]. These results suggest that PCNA and CAF-1 complex may respond synergistically to DNA damage. Interestingly, previous studies revealed that CHAF1A contains two PIP domains by sequence alignment, which may mediate the interaction between CHAF1A and PCNA [38]. Nevertheless, previous studies have shown that CHAF1A interacts with PCNA depending on which PIP domain is controversial [39]. Therefore, in this study, we demonstrated that CHAF1A interacts with PCNA through the PIP2 domain. Here, we found that CHAF1A promotes the TLS pathway in response to DNA replication stress by regulating PCNA K164 monoubiquitination. Unexpectedly, our data showed that CHAF1A promotes PCNA K164 monoubiquitination independent of the interaction between CHAF1A and PCNA. These results suggest that the involvement of CHAF1A in PCNA monoubiquitination is independent of its traditional histone assembly function.

The key steps in the TLS pathway are PCNA K164 monoubiquitination-mediated replacement of DNA polymerases and subsequent reactivation of DNA replication forks. In this study, we found that CHAF1A knockdown significantly inhibited the restart and elongation rate of DNA replication forks after UV radiation and HU treatment. CHAF1A knockdown significantly inhibited the recruitment of all Y-family TLS polymerases, suggesting that CHAF1A regulation of the TLS pathway appears to occur only upstream, namely PCNA K164 monoubiquitination. Moreover, CHAF1A did not affect the activation of RPA/ATR/CHK1 signaling pathway, further indicating that CHAF1A only plays a role in the activation of TLS pathway.

RAD6/RAD18 is a pair of E2/E3 ubiquitin ligases that mediate monoubiquitination of PCNA at K164 [40]. Although there are approximately 10 E2 enzymes and more than 60 E3 ligases in eukaryotes, RAD6/RAD18 is the major E2/E3 pair responsible for PCNA K164 monoubiquitination during TLS [40]. In our study, we demonstrated that CHAF1A promotes PCNA K164 monoubiquitination in a RAD18-dependent manner. Moreover, CHAF1A enhances the recruitment of RAD18 to PCNA under UV radiation. Here, through His pull-down and ssDNA/dsDNA pull-down assays, we found that CHAF1A mediated the recruitment of RAD18 on the stalled replication fork through direct interaction with RAD18. These results suggest that CHAF1A alone can directly mediate the recruitment of RAD18 at the stalled replication fork. Several proteins are known to be involved in RAD18 recruitment to the stalled replication fork to promote PCNA K164 monoubiquitination [43, 46–48]. In this study, we found that recombinant RAD18 also possess DNA-binding function. Therefore, we believe that RAD18 recruitment to the stalled replication fork is performed by a number of proteins, including CHAF1A, to enable cancer cells to rapidly respond to DNA replication stress.

The two subunits CHAF1A and CHAF1B of the CAF1 complex are often considered essential components for its histone assembly function. The absence of either can disrupt histone recruitment at the injury site [41]. Moreover, previous studies have found that the absence of CHAF1B does not affect the recruitment of CHAF1A at the UV damage site, but its molecular mechanism remains unclear [41]. This study found that CHAF1A knockdown significantly inhibited RAD18 recruitment and PCNA K164 monoubiquitination, while CHAF1B knockdown had no effect on RAD18 recruitment and subsequent PCNA K164 monoubiquitination. These results suggest that CHAF1A regulates TLS pathways not in the form of CAF1 complexes, but in the form of monomers. In addition, CHAF1B knockdown hindered the recruitment of histone H3.1 but did not affect the recruitment of RAD18, indicating that CHAF1A-mediated recruitment of RAD18 at the stalled replication fork was not related to the recruitment of histone H3.1.

In summary, our study identified CHAF1A as a key regulator of TLS pathway in cancer cells and CHAF1A promotes cancer cell survive by regulating the TLS pathway. CHAF1A promotes fork restart under DNA damage. Mechanistically, we demonstrated that CHAF1A promotes PCNA K164 monoubiquitination in a RAD18-dependent manner, and this process is independent of CHAF1A-PCNA interaction. We found that CHAF1A regulates the TLS pathway independently of its histone assembly function. Moreover, we revealed that CHAF1A directly interacts with RAD18, mediating RAD18 recruitment to stalled replication forks under DNA damage. Together, the findings of this study enrich our understanding of the regulatory mechanisms underlying the TLS pathway.

## Supplementary information

Supplementary Figure and Figure Legends

Full and uncropped western blots

## Data Availability

The data underlying this article are available in the article and in its online supplementary material.
