# Supplementary material for: Chromatin assembly factor 1 subunit A promotes TLS pathway by recruiting E3 ubiquitin ligase RAD18 in cancer cells
Source: Cell Death Dis. 2025 Mar 1;16(1):147. doi: 10.1038/s41419-025-07468-5 (PMC11873243; doi:10.1038/s41419-025-07468-5)
Supplement: Supplementary file 1 — Supplementary Figure and Figure Legends [file 41419_2025_7468_MOESM1_ESM.docx]

**Chromatin assembly factor 1 subunit A promotes TLS pathway by recruiting E3 ubiquitin ligase RAD18 in cancer cells**

Bing Wen ^1^, Hai-Xiang Zheng ^2^, Jing-Hua Heng ^2^, Qian Tang ^1^, Dan-Xia Deng ^2^, Zhi-Da Zhang ^1^, Lian-Di Liao ^1^, Li-Yan Xu ^1,2*^ and En-Min Li ^1,2,3*^

^1^ The Key Laboratory of Molecular Biology for the High Cancer Incidence Coastal Chaoshan Area, Department of Biochemistry and Molecular Biology, Shantou University Medical College, Shantou 515041, Guangdong, P.R. China

^2^ Chaoshan Branch of State Key Laboratory for Esophageal Cancer Prevention and Treatment, Institute of Oncologic Pathology, Shantou University Medical College, Shantou 515041, Guangdong, P.R. China

^3^ The Laboratory for Cancer Molecular Biology, Shantou Academy of Medical Sciences, Shantou 515041, Guangdong, P.R. China.

*Corresponding authors: Dr. En-Min Li, Department of Biochemistry and Molecular Biology, Shantou University Medical College, 22 Xinling Road, Shantou 515041, P.R. China. Phone: 86-754-88900413, Fax: 86-754-88900847, E-mail: nmli@stu.edu.cn. Correspondence may also be addressed to Dr. Li-Yan Xu (E-mail: [lyxu@stu.edu.cn](mailto:lyxu@stu.edu.cn))

**Supplementary Figures:**

**Supplementary Figure 1. Knockdown of CHAF1B does not affect UV-induced PCNA K164 monoubiquitination.**

**Supplementary Figure 2. Knockdown of CHAF1A inhibits HU-induced PCNA K164 monoubiquitination.**

**Supplementary Figure 3. CHAF1A promotes the TLS pathway induced by HU.**

**Supplementary Figure 4. CHAF1A reduces DNA damage induced by DNA replication stress, but does not affect RPA-ATR-CHK1 signaling pathway.**

**Supplementary Figure 5. CHAF1A, not CHAF1B, mediates RAD18 recruitment.**

**Supplementary Figure 6. ssDNA/dsDNA pull down assay.**


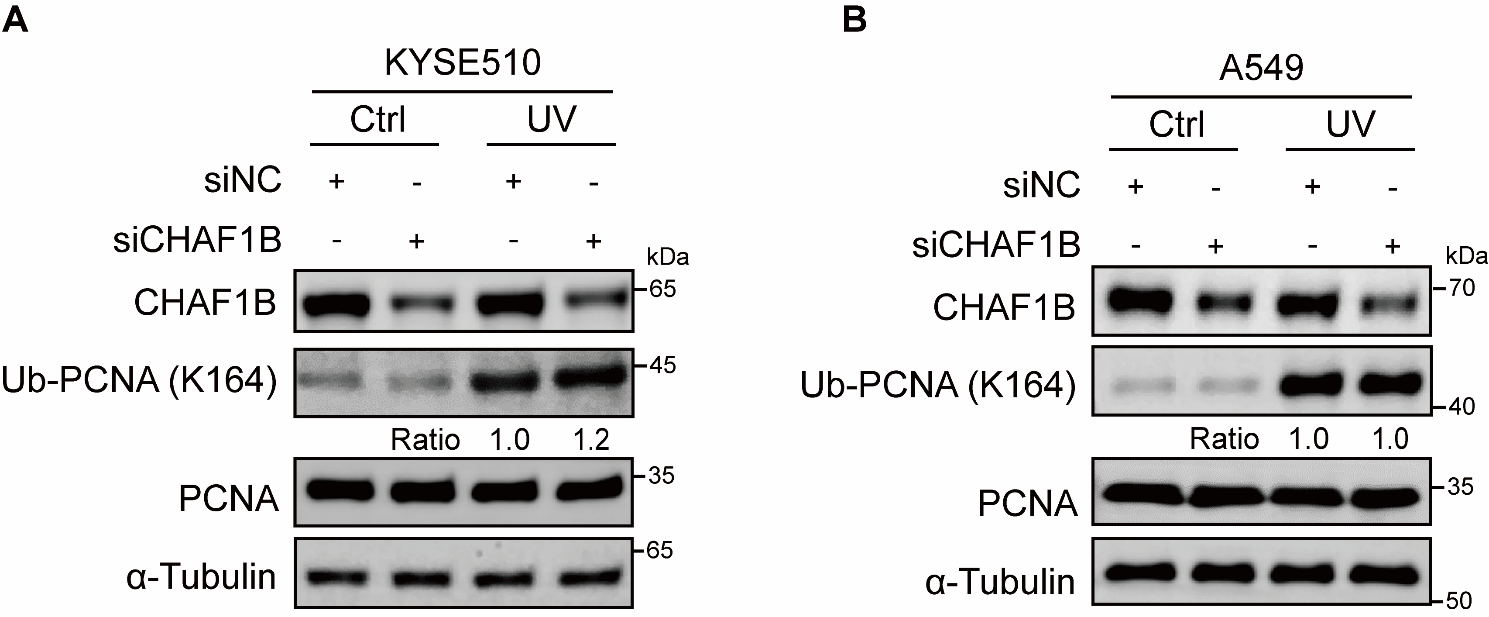


**Supplementary Figure 1. Knockdown of CHAF1B does not affect UV-induced PCNA K164 monoubiquitination.** (A and B) KYSE510 cells or A549 cells were transfected with control or CHAF1B siRNA oligos for 24 h and then treated with or without 100 J/m^2^ UV for 1h. Whole-cell lysates were analyzed using western blotting with the indicated antibodies.


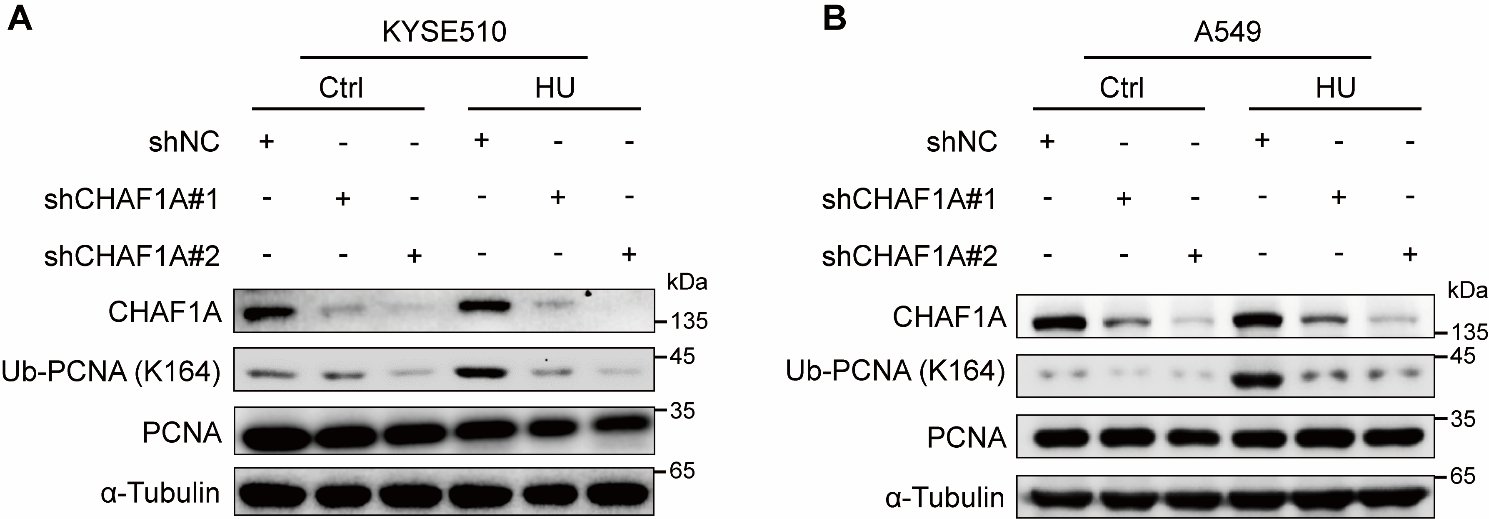


**Supplementary Figure 2. Knockdown of CHAF1A inhibits HU-induced PCNA K164 monoubiquitination.**

(A and B) CHAF1A knockdown KYSE510 cells or A549 cells were treated with or without 2mM HU for 4 h. Whole-cell lysates were analyzed using western blotting with the indicated antibodies.

**
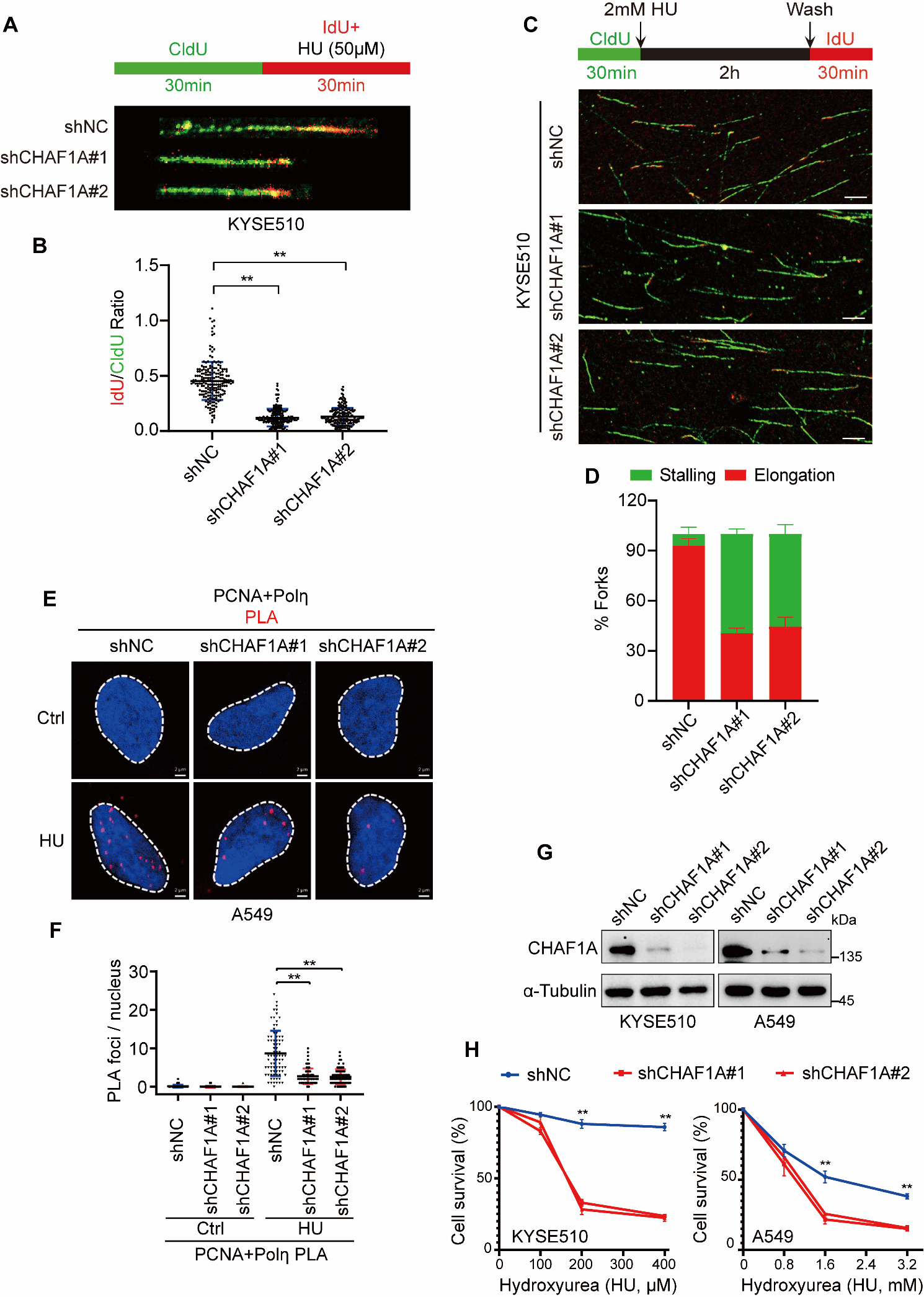
**

**Supplementary Figure 3. CHAF1A promotes the TLS pathway induced by HU.**

(A) DNA fiber assay was used to measure DNA replication in inducible CHAF1A knockdown KYSE510 cells. Schematic of alternative CldU/IdU pulse-labeling protocol to evaluate fork status. Representative images of DNA fiber assays are shown. Data are plotted in (B) (n = 200). Data are representative of at least three independent experiments. ^**^*P* < 0.01; one-way ANOVA. (C) Schematic of alternative CldU/IdU pulse-labeling protocol to evaluate fork status. Representative images of DNA fiber assays are shown. (D) Image J was used to calculate the percentage of stalling forks and elongation forks in each field of view (n = 10). Data represent means ± SEM from three independent experiments. ^**^*P* < 0.01; one-way ANOVA. (E) CHAF1A knockdown A549 cells were treated with or without 2mM HU for 4 h. PLA assays was used to detect the interaction between PCNA and Pol η (scale bar, 2 μm), and (F) each spot represents the number of PLA foci in an individual nucleus (n = 80). Data represent means ± SEM from three independent experiments. ^**^*P* < 0.01; one-way ANOVA. (G) The CHAF1A knockdown in KYSE510 cells and A549 cells was detected by western blotting. (H) Statistical plots of colony formation assay (n = 3). Data represent the mean ± SEM from three independent experiments. ^*^*P* < 0.05; one-way ANOVA.

**
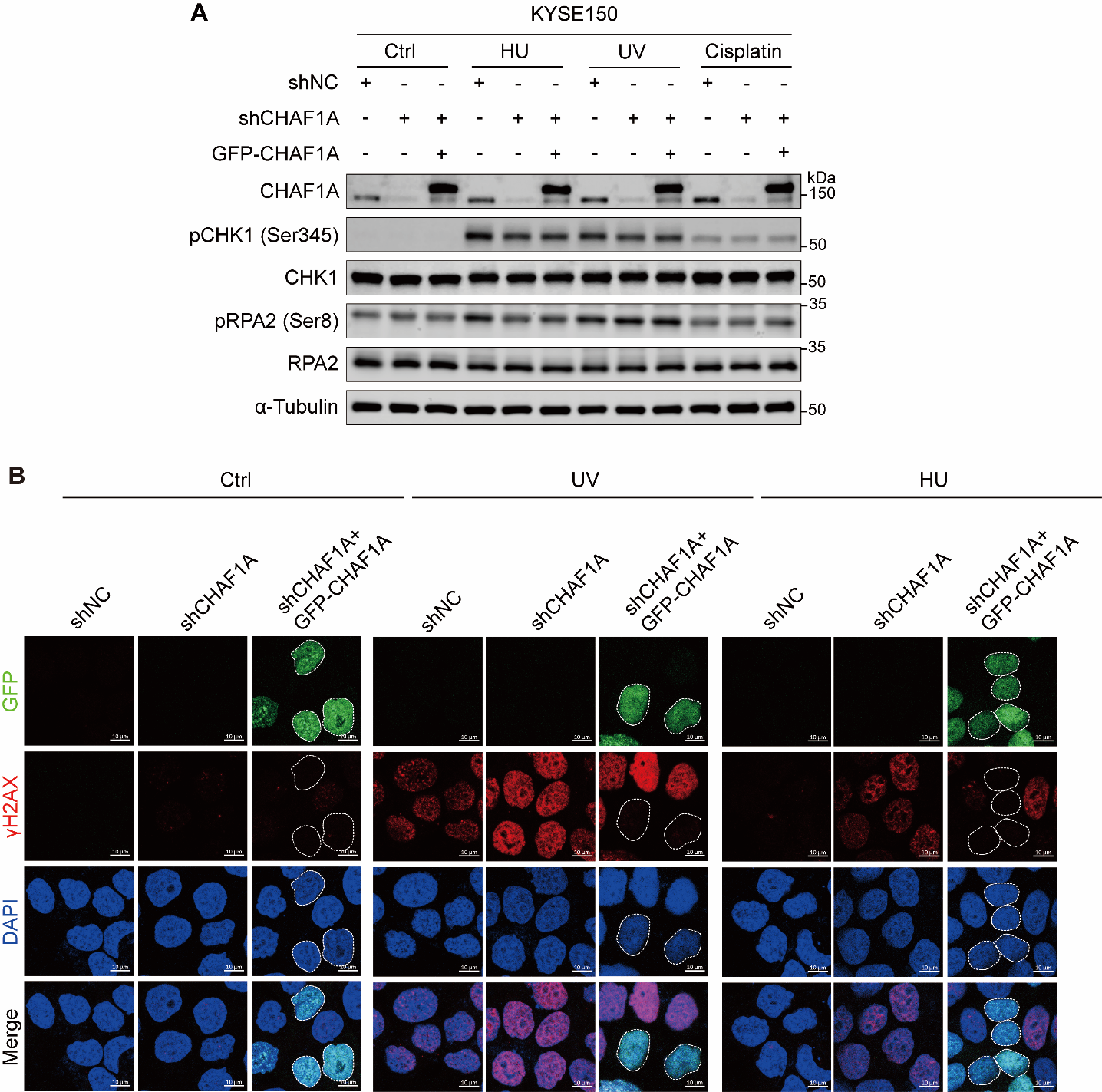
**

**Supplementary Figure 4. CHAF1A reduces DNA damage induced by DNA replication stress, but does not affect RPA-ATR-CHK1 signaling pathway.**

(A) CHAF1A-knockdown KYSE150 cells were transfected with plasmid encoding GFP-CHAF1A and then treated with HU (2mM 4h), UV (100 J/m^2^ 1h) or cisplatin (30μM 6h). Whole-cell lysates were analyzed using western blotting with the indicated antibodies. (B) CHAF1A-knockdown KYSE150 cells were transfected with plasmid encoding GFP-CHAF1A and then treated with HU (2mM 4h) or UV (100 J/m^2^ 1h), and then release to 12h. Immunofluorescence was used to detect GFP-CHAF1A, γH2AX or DAPI (scale bar, 10 μm).


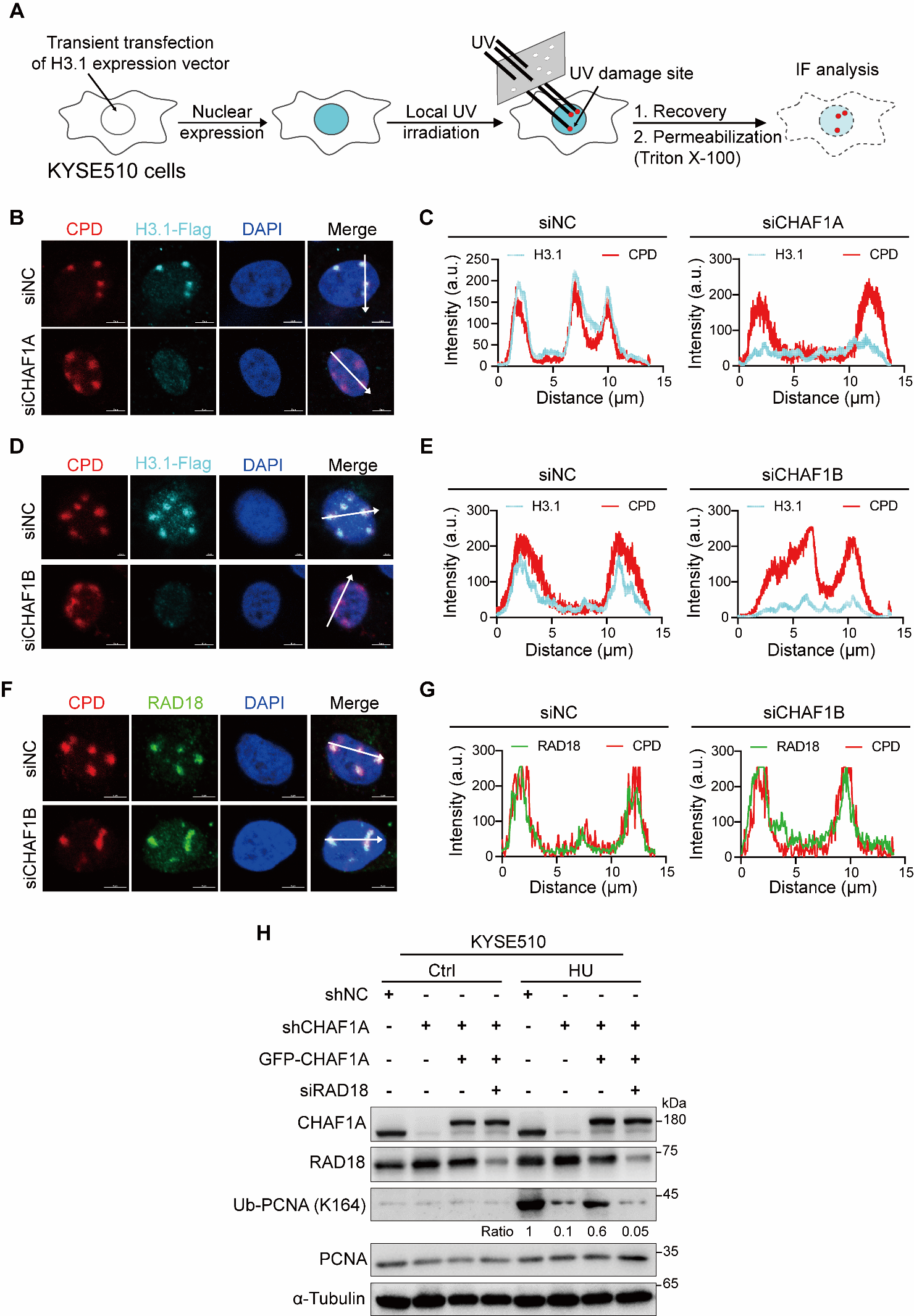


**Supplementary Figure 5. CHAF1A, not CHAF1B, mediates RAD18 recruitment.**

(A) Experimental scheme for UV damage induction in spots. To analyze the behavior of Histone H3.1 after UV damage, we transfected a Flag-labeled H3.1 vector into KYSE510 cells, then, we selected and micro-irradiated with 100 J/m^2^ in KYSE510 cells. (B)(D) After UV damage, the cells underwent immunofluorescence (IF) analysis. (C)(E) ZEN software was used to calculate the fluorescence intensity of H3.1 and CPD on the white arrow. (F) RAD18 recruitment after knocking down CHAF1B was detected by micro-radiation in KYSE510 cells. (G) ZEN software was used to calculate the fluorescence intensity of RAD18 on the white arrow. (H) CHAF1A-knockdown KYSE510 cells were transfected with indicated siRNAs before transfection with plasmid encoding GFP-CHAF1A and then treated with or without 2mM HU for 4 h. Whole-cell lysates were analyzed using western blotting with the indicated antibodies.


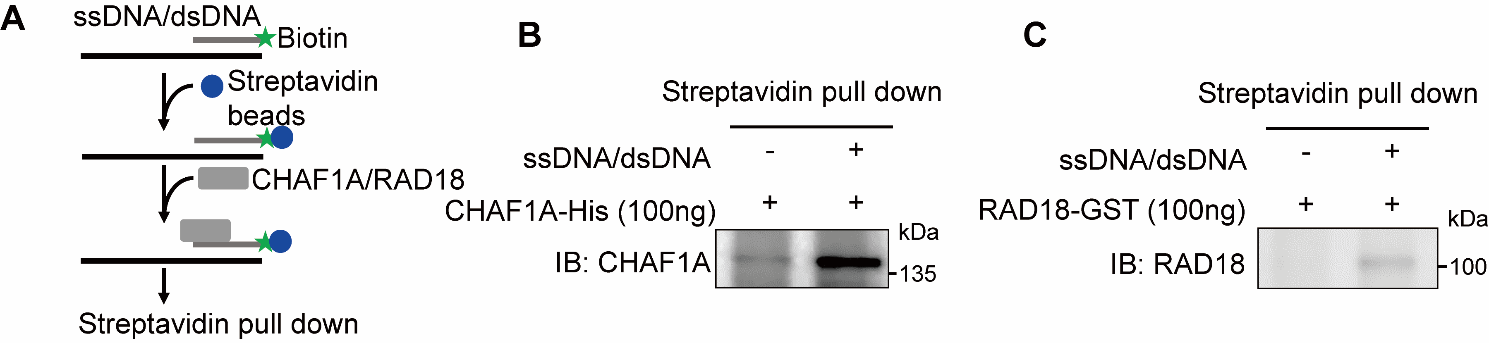


**Supplementary Figure 6. ssDNA/dsDNA pull down assay.**

(A) Schematic of ssDNA/dsDNA pull-down assay. (B) Biotinylated ssDNA/dsDNA were first bound with streptavidin beads and coated with bacterially produced recombinant CHAF1A-His, followed by immunoblotting with CHAF1A antibody. (C) Biotinylated ssDNA/dsDNA were first bound with streptavidin beads and coated with bacterially produced recombinant RAD18-GST, followed by immunoblotting with RAD18 antibody.
