# Supplementary material for: Chromatin assembly factor 1 subunit A promotes TLS pathway by recruiting E3 ubiquitin ligase RAD18 in cancer cells
Source: Cell Death Dis. 2025 Mar 1;16(1):147. doi: 10.1038/s41419-025-07468-5 (PMC11873243; doi:10.1038/s41419-025-07468-5)
Supplement: Supplementary file 2 — Full and uncropped western blots [file 41419_2025_7468_MOESM2_ESM.pdf]

Figure 1

Figure 1A

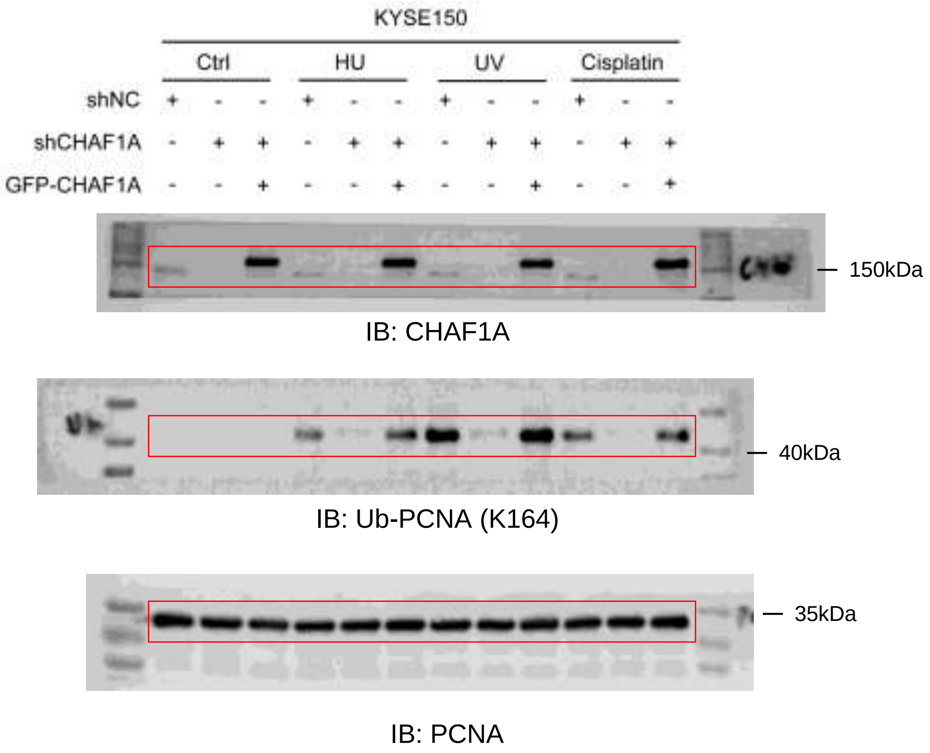

Figure 1

Figure 1B Figure 1C

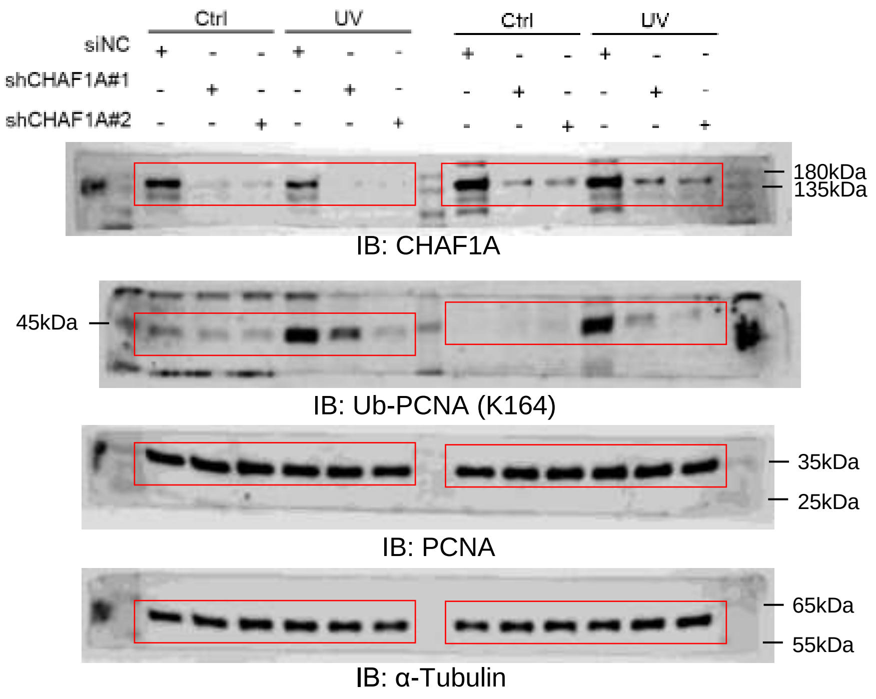

Figure 1D

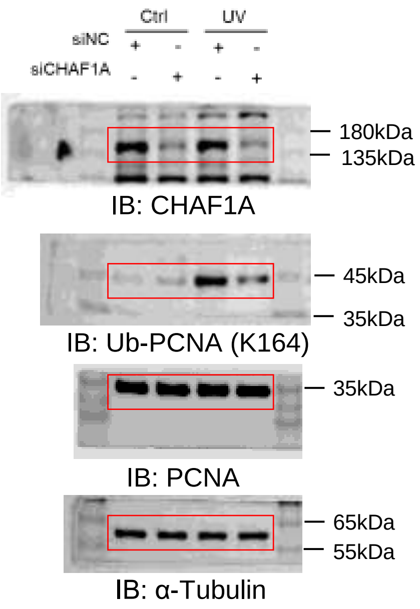

Figure 1E

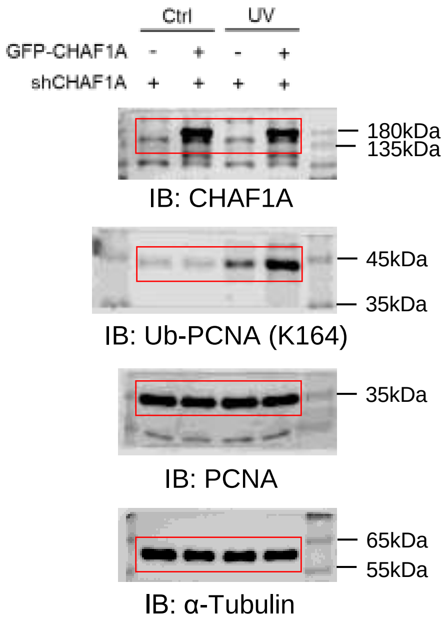

Figure 1

Figure 1F

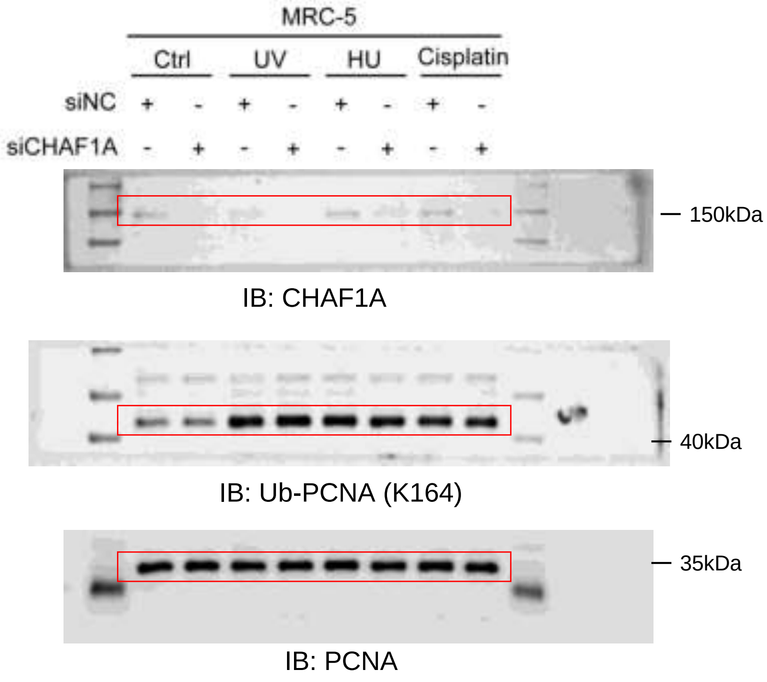

Figure 1G

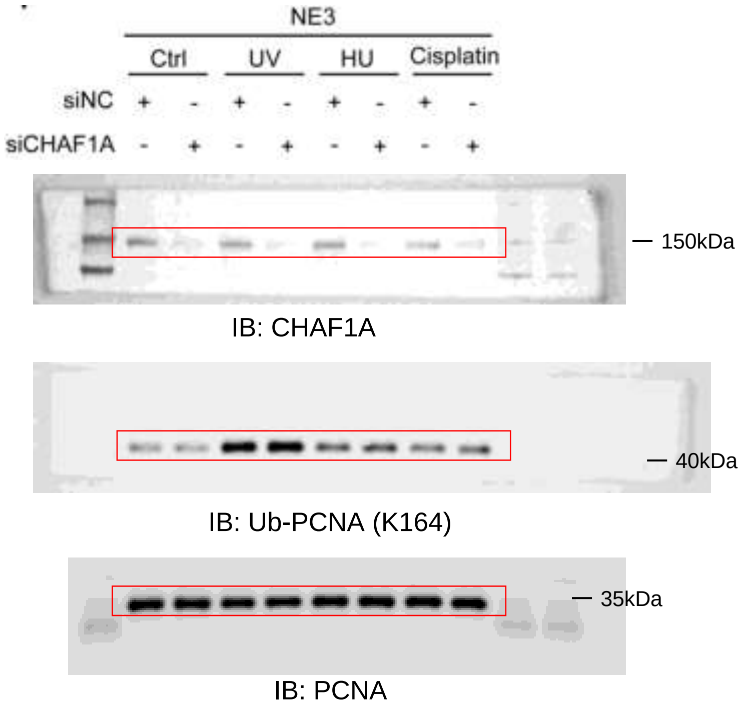

Figure 2

Figure 2E

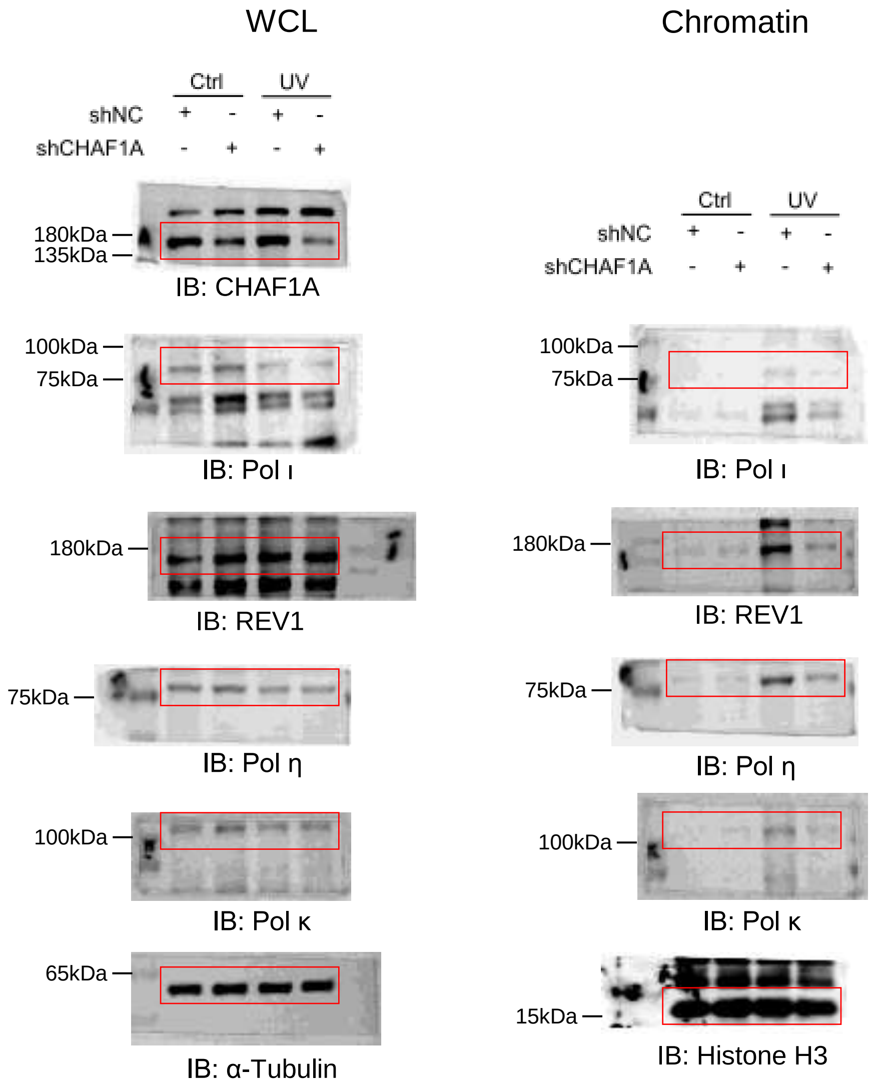

Figure 3

Figure 3B

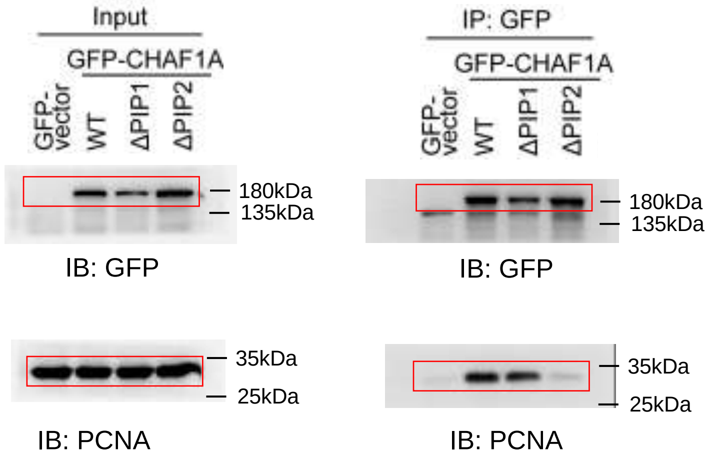

Figure 3C

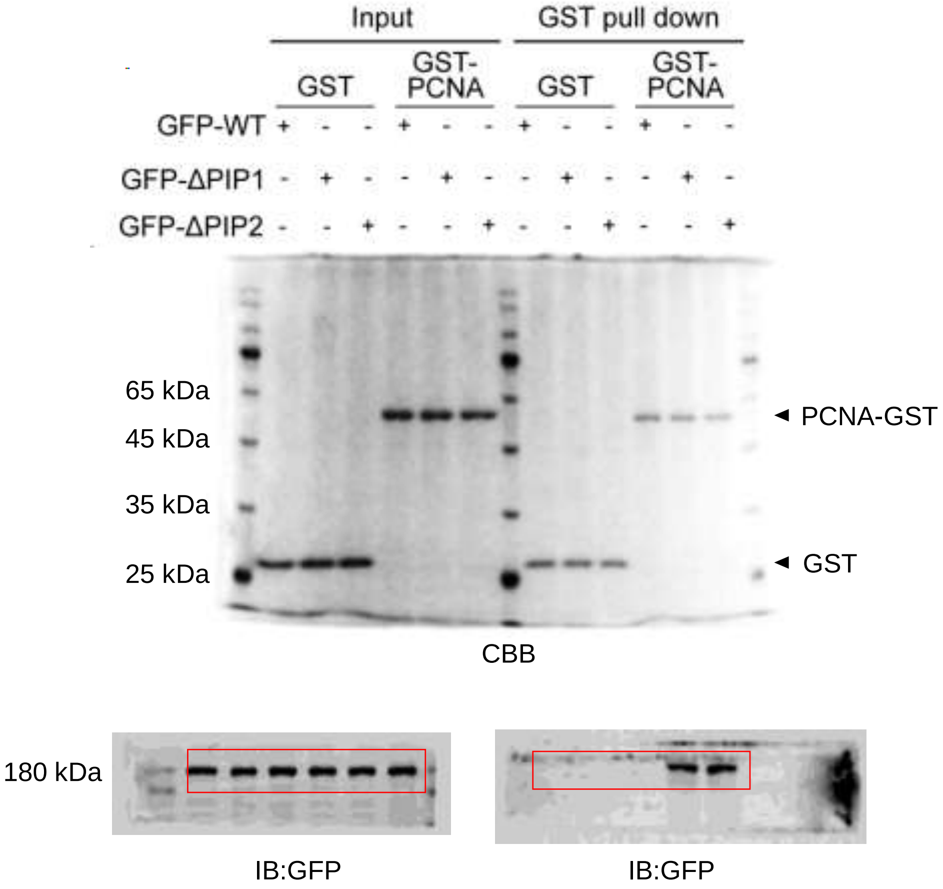

Figure 3

Figure 3F

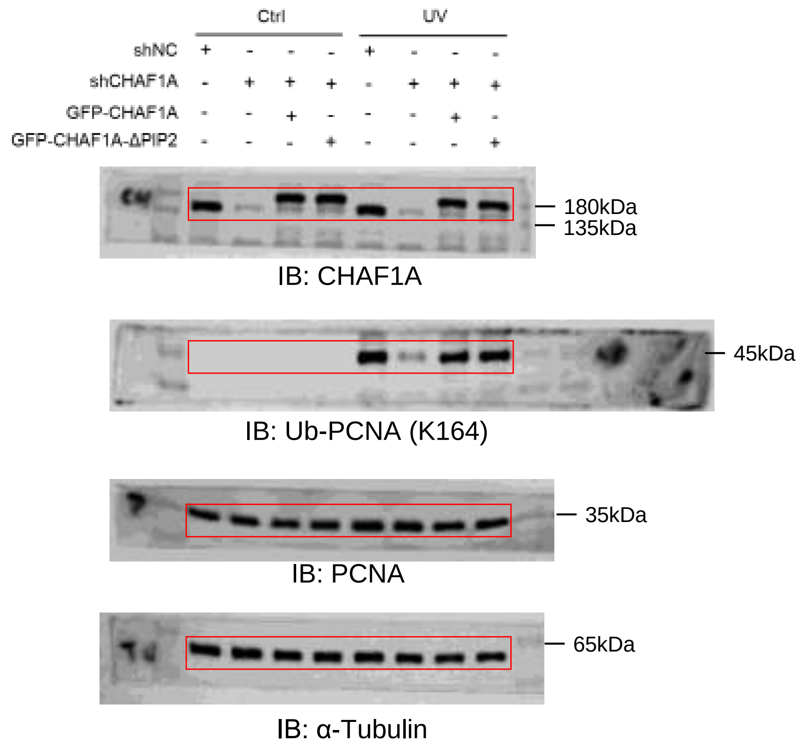

Figure 3G

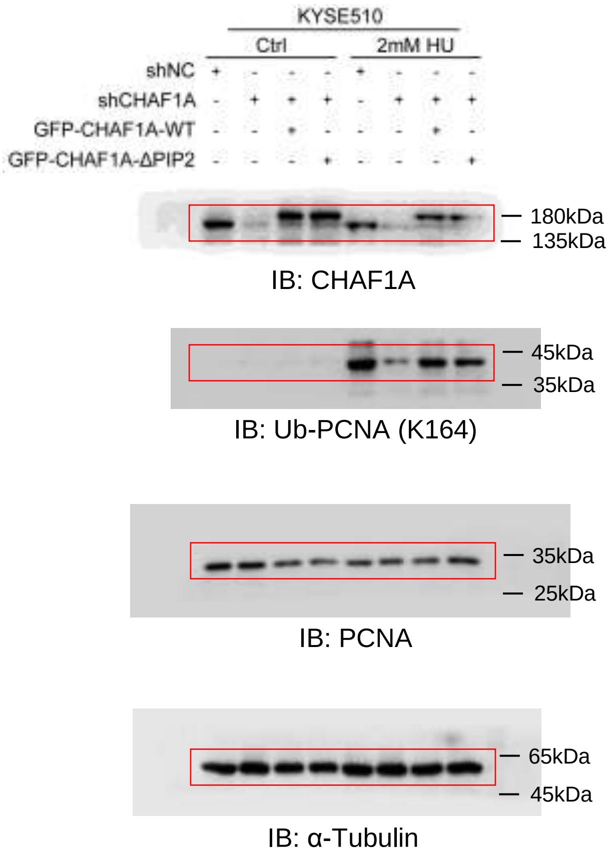

Figure 4

Figure 4A

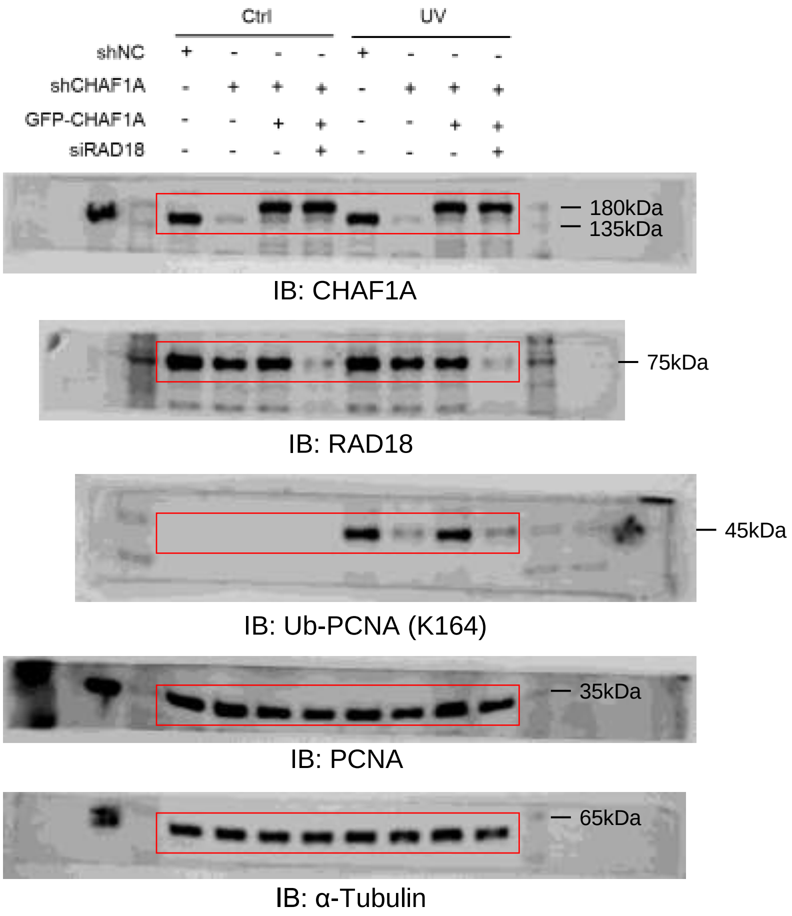

Figure 4F

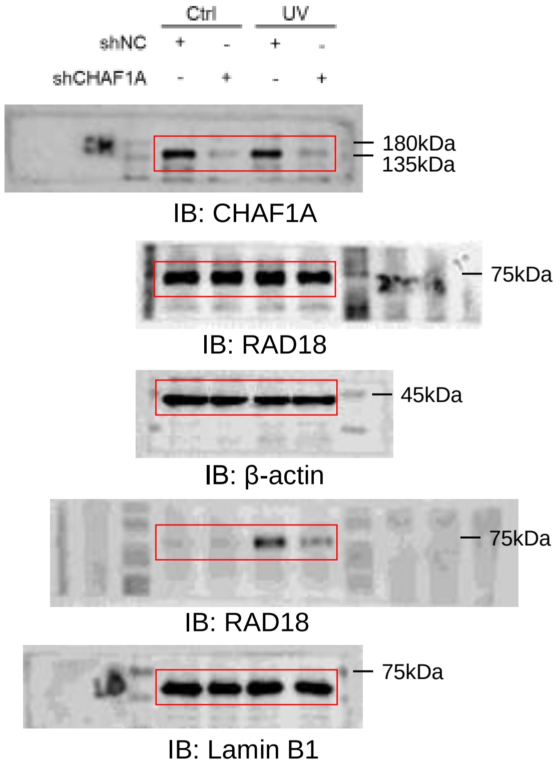

Figure 5

Figure 5A

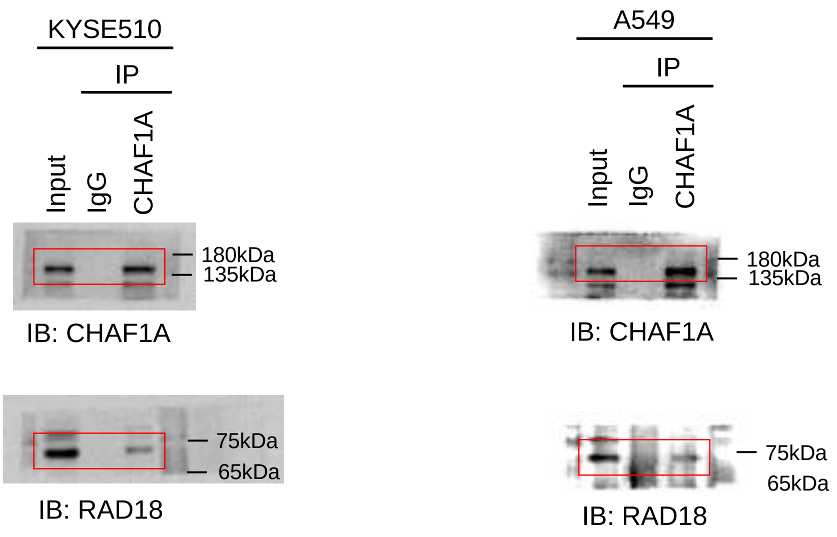

Figure 5C

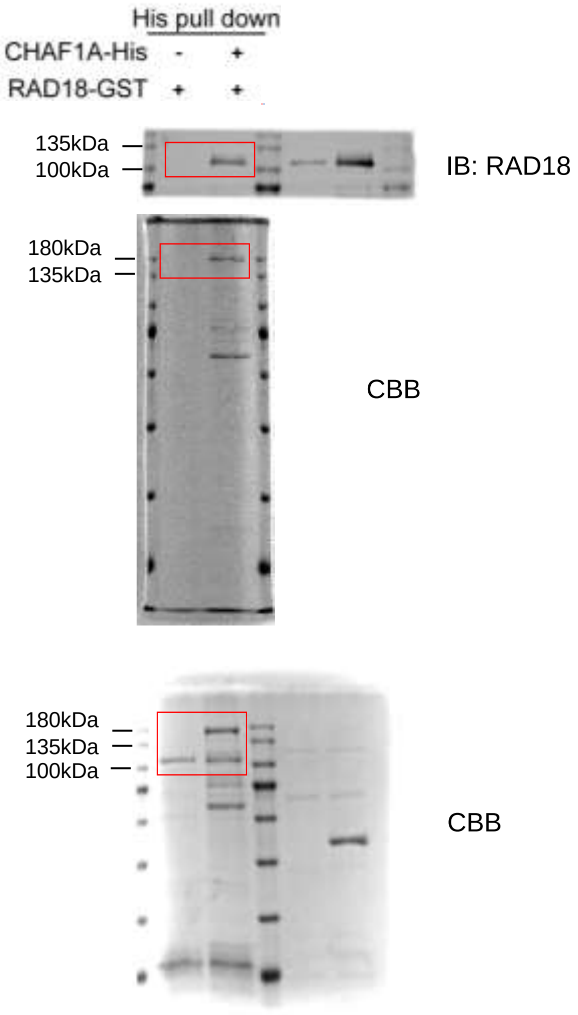

Figure 5

Figure 5E

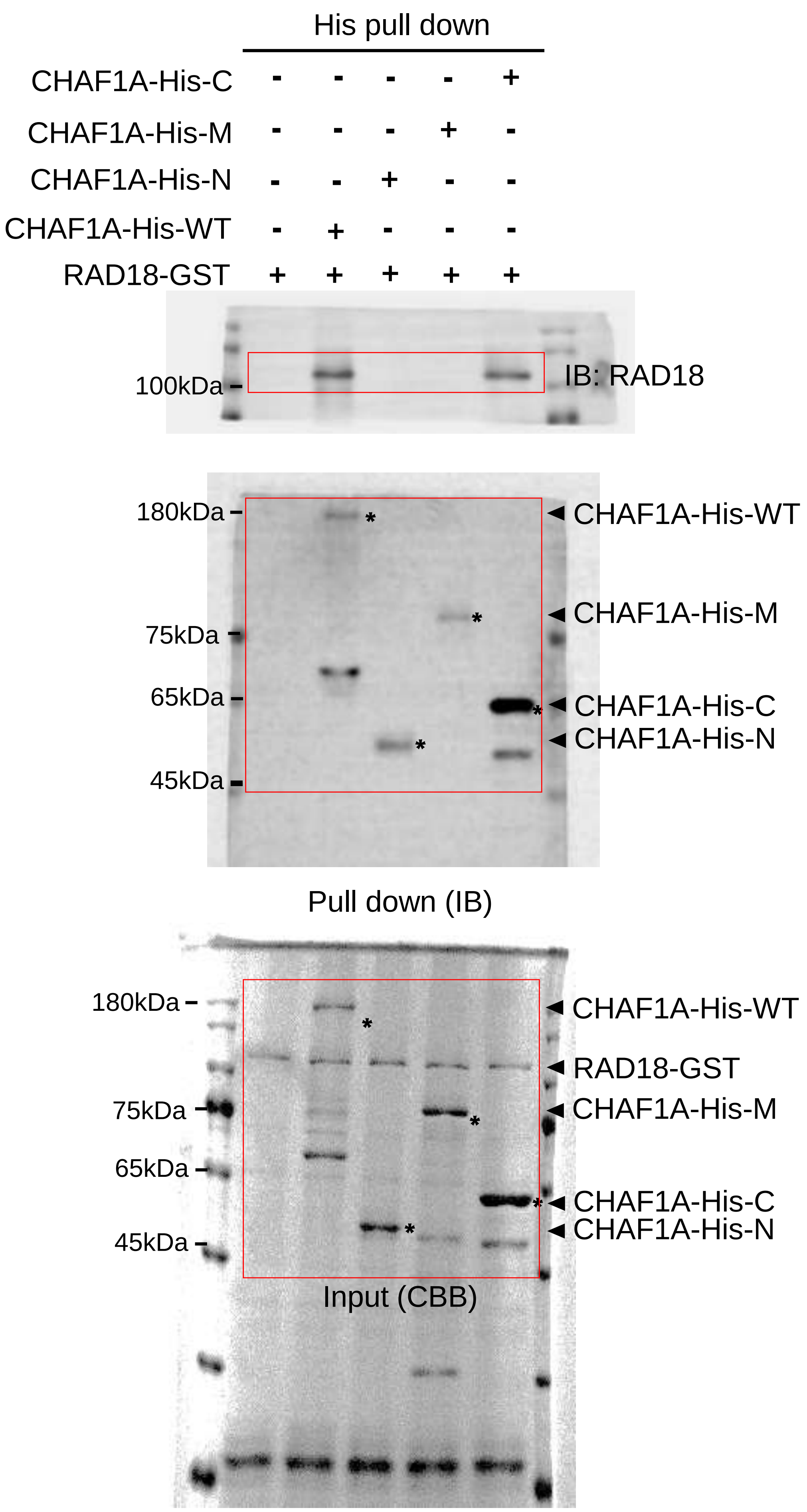

Figure 6

Figure 6E

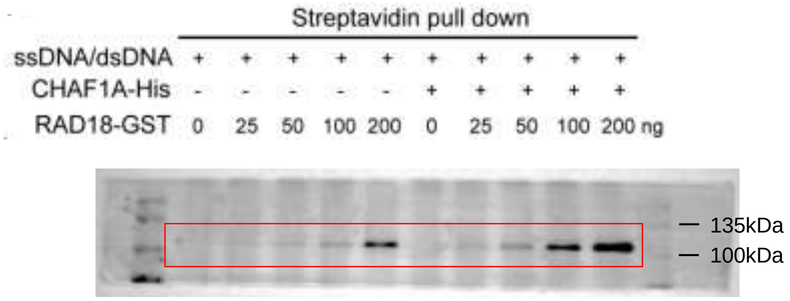

IB: RAD18

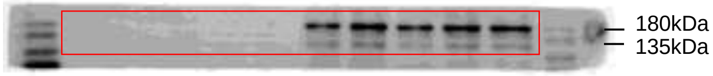

IB: CHAF1A

Figure 6

Figure 6G

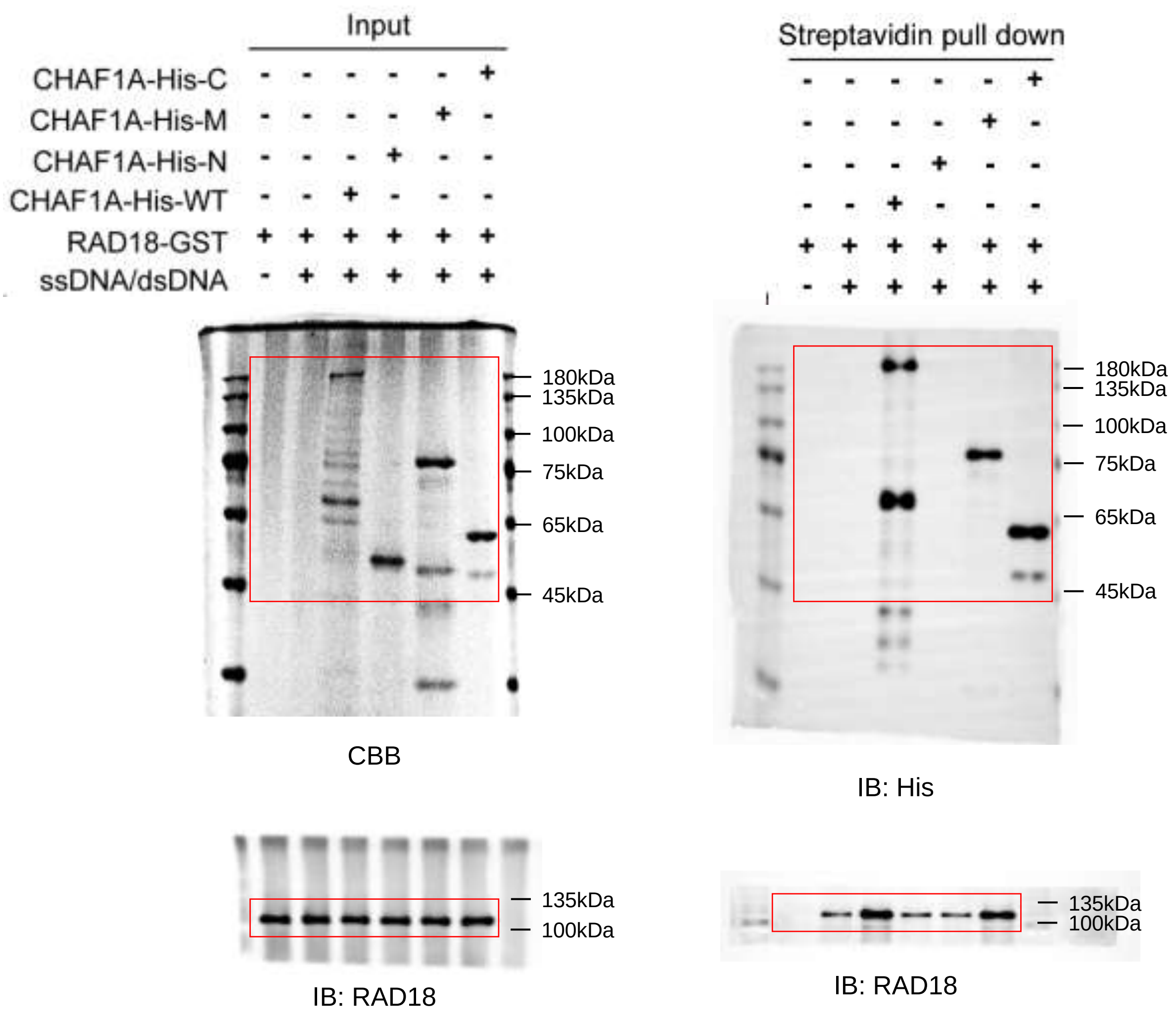

Figure 6

Figure 6I

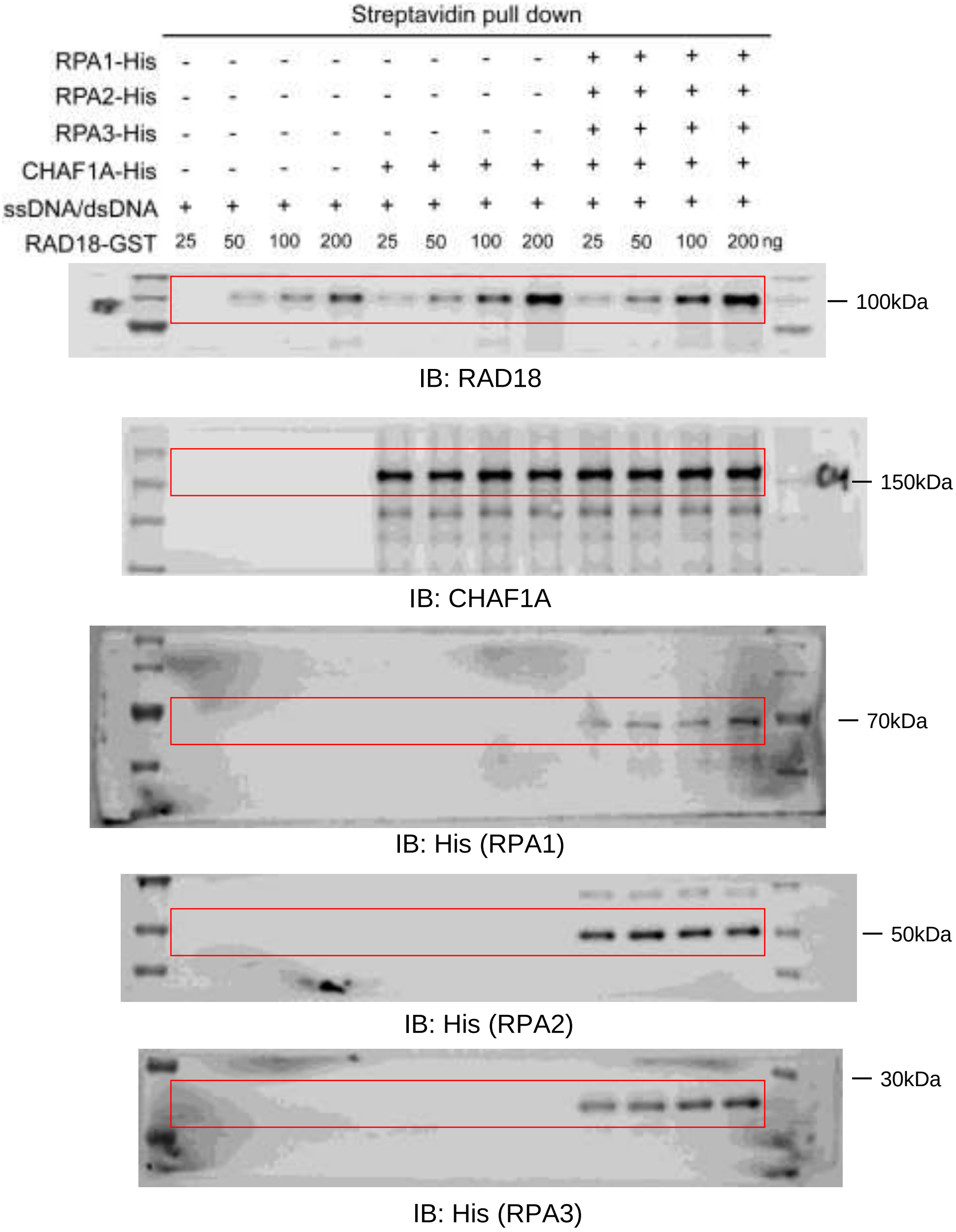

Figure 7

Figure 7B

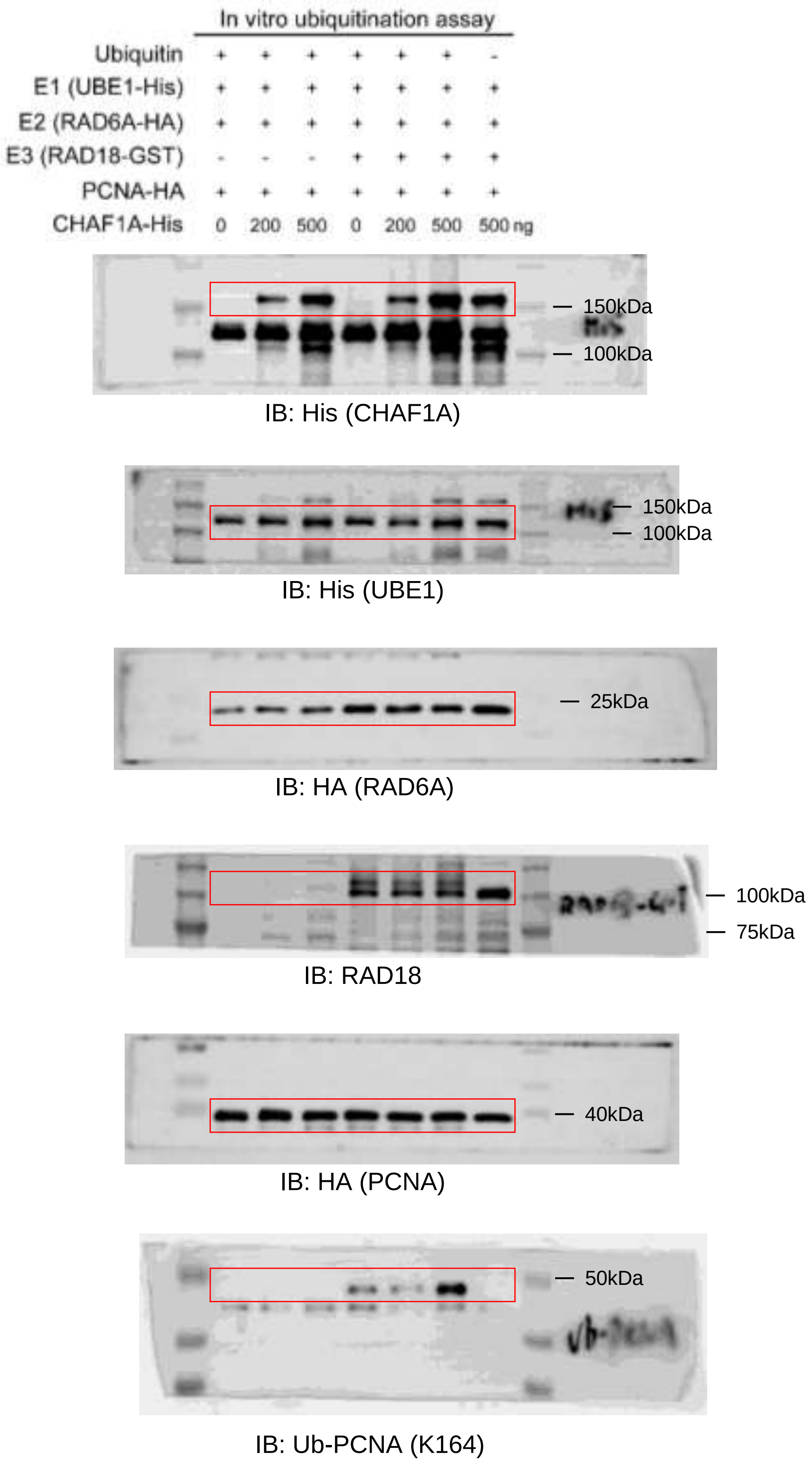

Supplementary Figure 1

Supplementary Figure 1A

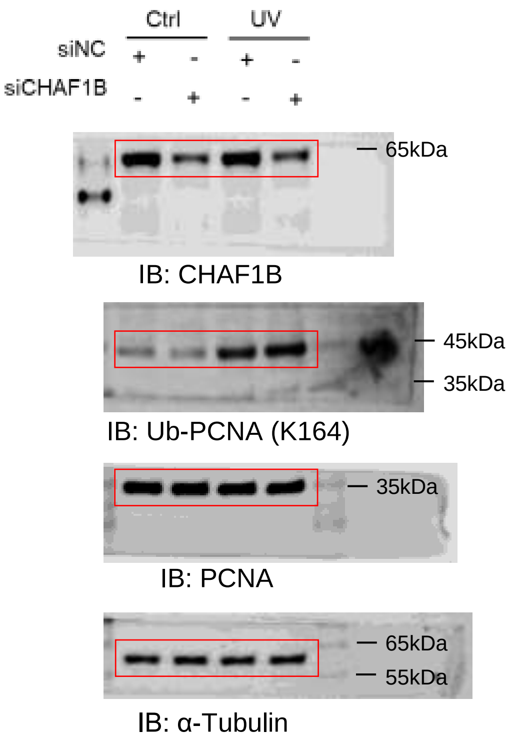

Supplementary Figure 1B

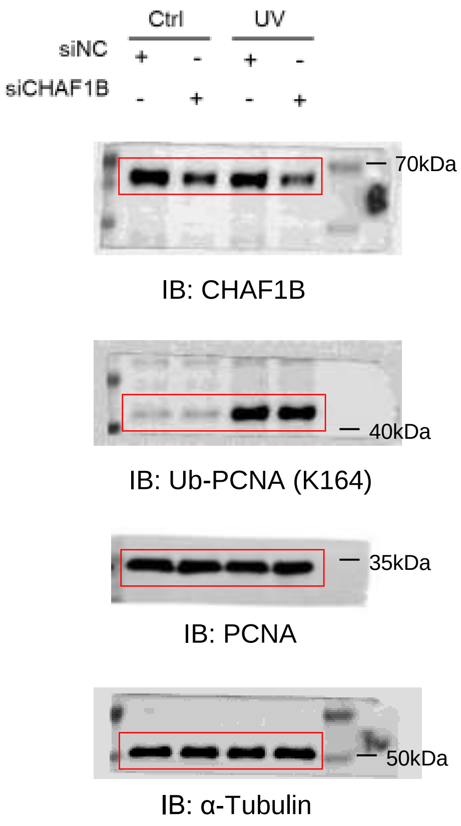

Supplementary Figure 2

Supplementary Figure 2A

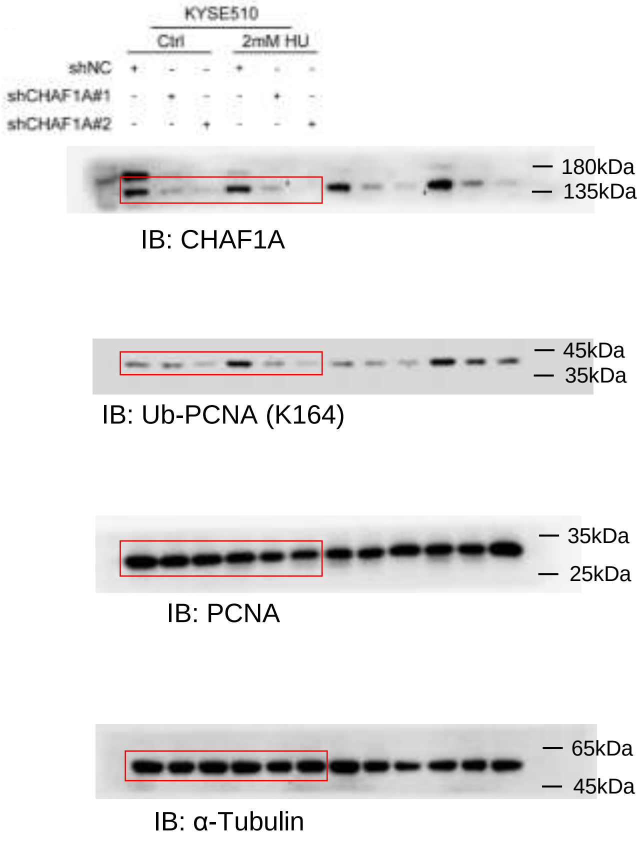

Supplementary Figure 2B

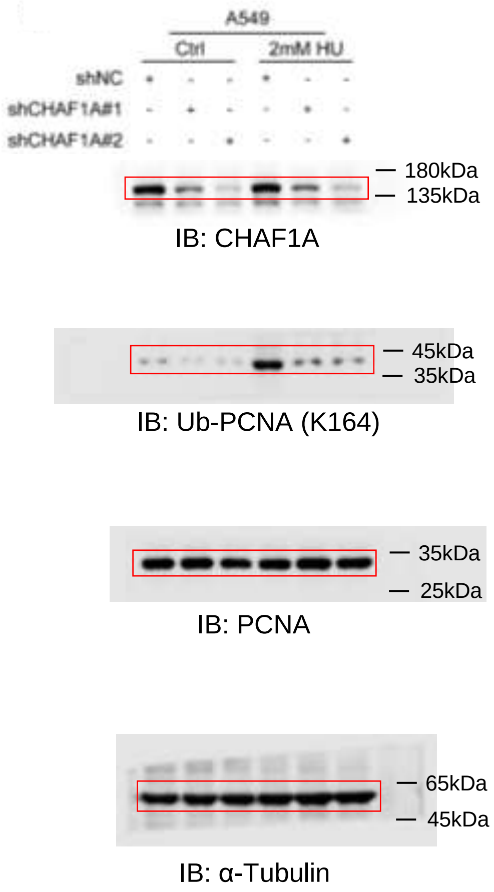

Supplementary Figure 3

Supplementary Figure 3G

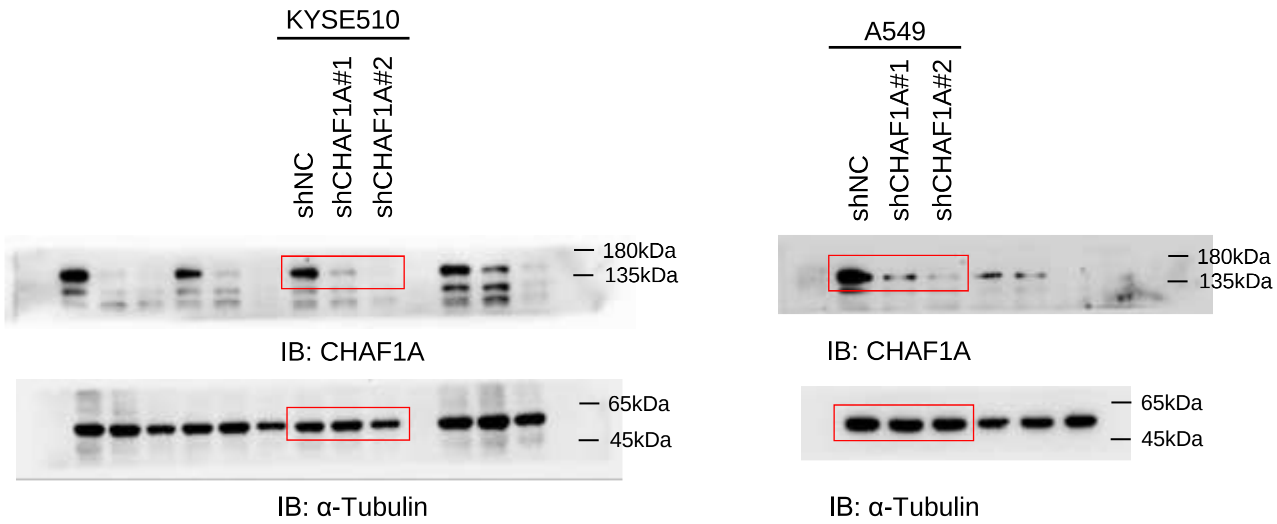

Supplementary Figure 4

Supplementary Figure 4A

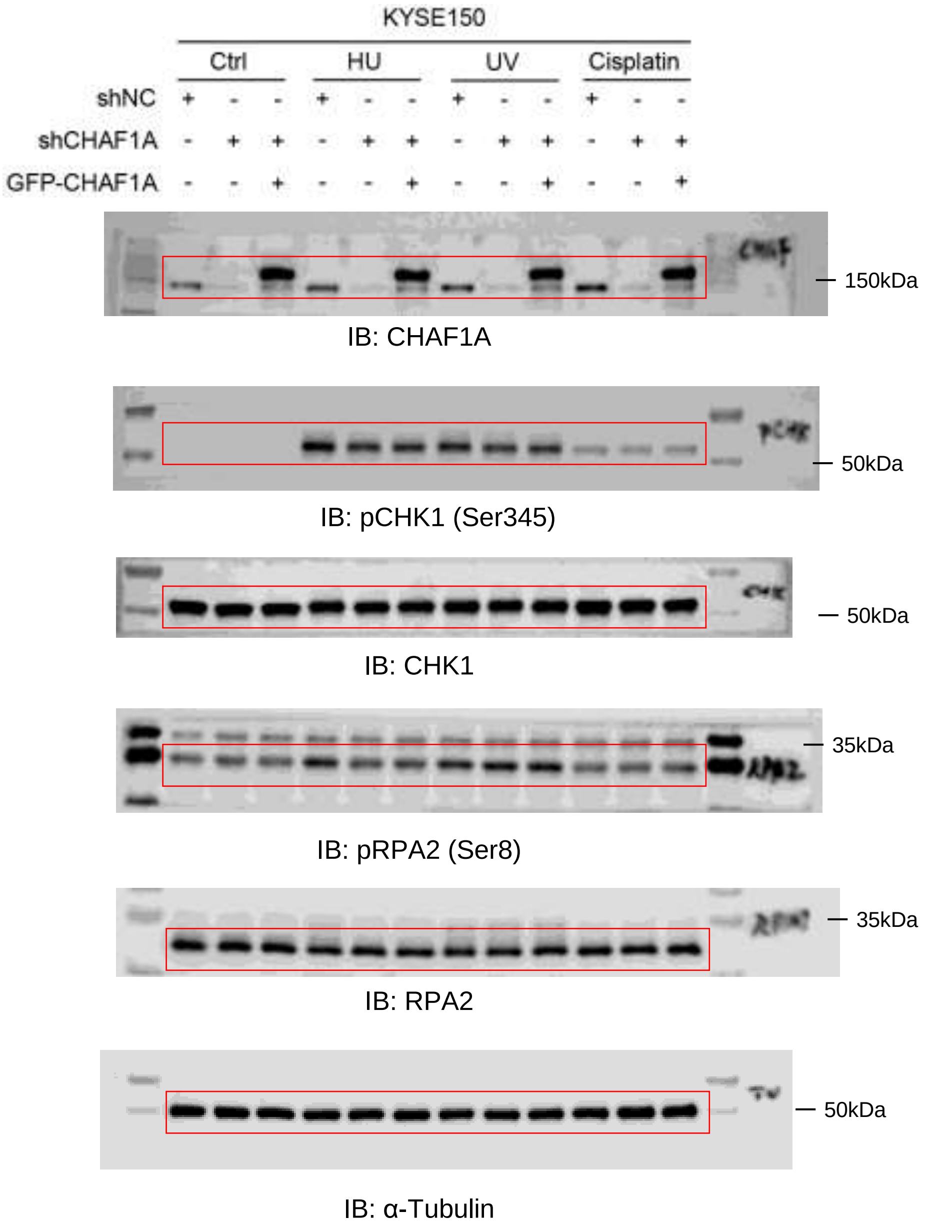

Supplementary Figure 5

Supplementary Figure 5H

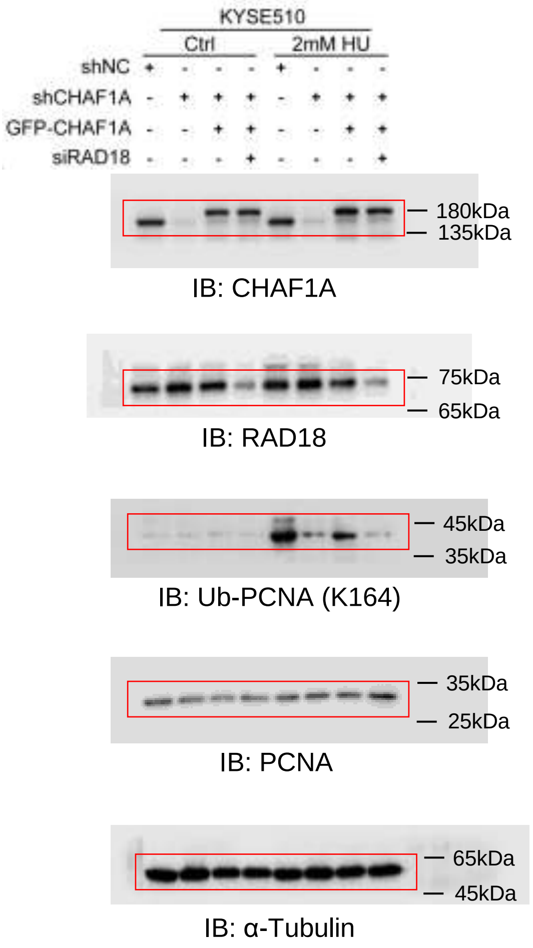

Supplementary Figure 6

Supplementary Figure 6B

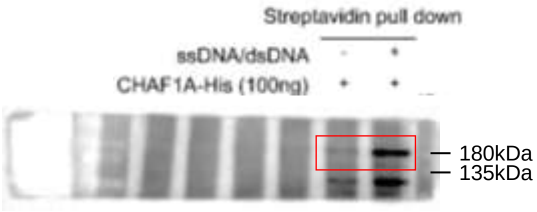

IB: CHAF1A

Supplementary Figure 6C

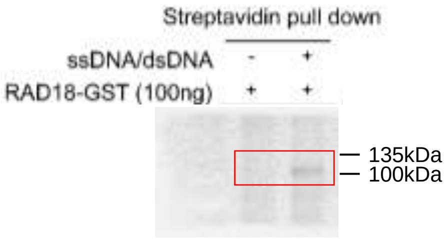

IB: RAD18
